# Discovering the diversity of tadpoles in the mid-north Brazil: morphological and molecular identification, and characterization of the habitat

**DOI:** 10.7717/peerj.16640

**Published:** 2023-12-14

**Authors:** Patricia dos Santos Sousa, Carlos Augusto Silva Azevedo, Maria Claudene Barros, Elmary Costa Fraga, Thaís B. Guedes

**Affiliations:** 1 Centro de Estudos Superiores de Caxias, Universidade Estadual do Maranhão, Caxias, Maranhão, Brazil; 2 Departamento de Biologia Animal, Instituto de Biologia, Universidade Estadual de Campinas, Campinas, SP, Brazil; 3 Gothenburg Global Biodiversity Center, University of Gothenburg, Gothenburg, Sweden

**Keywords:** Babaçu Forest of Maranhão, Biodiversity, Cerrado, Conservation, Geographic distribution, Tadpole habitats, Tadpole morphology, Tadpole oral morphology, Taxonomy

## Abstract

Brazil stands out for presenting the highest amphibian anuran diversity in the world. However, taxonomic studies that address characteristic of larval stage of anurans are incipient, representing only 62% of known species. We assess the species diversity of tadpoles from eastern Maranhão state, mid-northern region of Brazil based on morphological and molecular identification (*i.e*., 16S rRNA gene fragment), and we also provide characteristics of the habitats occupied by each species. We carried out 30 field samplings during 13 months in 16 environments along an ecotonal area, over five cities inside the limits of state of Maranhão, between the Maranhão Babaçu Forest and Cerrado ecoregions. We searched for tadpoles in a variety of water bodies, and the tadpoles that reached the developmental stage between 34 to 40 Gosner were morphologically identified. The tadpoles collected herein represent 26 species belonging to five families. The external morphology enabled the identification of 24 species, while the molecular data recognized 22 unique evolutionary units. The most represented family was Hylidae (Hylinae 11 spp., Phyllomedusinae one spp.) followed by Leptodactylidae (Leptodactylinae seven spp., Leiuperinae three spp.), Microhylidae (Gastrophryninae two spp.), and Bufonidae (two spp.). Our results show that oral morphology was the most important character for identifying tadpoles based on morphology, and the specific 16S rRNA primer was suitable for molecular identification. This study pioneers the use of both morphological and molecular data to identify tadpoles in the state of Maranhão. It also provides, for the first-time, habitat characteristic for the species. Our study reveals a high number of anuran species sampled at the larval stage in the region, identifies species that require further taxonomic and systematic attention, and extends the geographic distribution of six species, three of which represent new occurrences for the state. Our results strengthen the hypothesis that the diversity of amphibians from Maranhão is underestimated and highlight the importance of herpetological inventories in poorly sampled areas, decentralizing the knowledge of biodiversity.

## Introduction

Brazil stands out for presenting the highest amphibian anuran diversity in the world, with 1,144 known species (Segalla et al., 2021) and the addition of new species and new occurrence records every year (*e.g*., Mângia et al., 2022; Ferrão et al., 2022). A unique feature to most anuran amphibians is the presence of a larval stage (tadpole), usually aquatic free-living and exotrophic, followed by a terrestrial stage; thus, the biphasic life cycle of anurans allows them to explore both aquatic and terrestrial environments (Haddad et al., 2013). However, much of the diversity of amphibians is known from adult specimens, with tadpoles’ morphological descriptions representing only 62% of known species in the country (Provete et al., 2012). Therefore, accessing tadpole diversity is important to advance the knowledge of this taxonomically and evolutionarily diverse group and provide crucial information for implementing effective conservation strategies (Nori et al., 2015; Pabijan et al., 2020). Furthermore, tadpoles are good models for ecological studies of community structure (Skelly, 1997; Hero, Gascon & Magnusson, 1998; Vera Candioti, 2007), indicating what kind of environments are preferable for developmental success (Kopp, Wachlevski & Eterovick, 2006; da Silva, Eskinazi-Sant’Anna & Silvério Pires, 2020). Finally, tadpoles are also relevant in studies investigating the decline in diversity of anuran communities caused by the fungus *Batrachochytrium dendrobatidis* (*e.g*., Ruggeri, Toledo & Carvalho-e-Silva, 2018).

In recent decades, there has been a considerable advance in the knowledge about the diversity of anurans in Brazil (*e.g*., Ramos et al., 2019; Silva-Alves et al., 2019; Segalla et al., 2021). However, the high morphological diversity, especially in the tropics, makes tadpoles the “Achilles heel”, *i.e*., the weakness of amphibian diversity studies (McDiarmid & Altig, 1999; Dubeux et al., 2020a, 2020b). In addition, tadpole studies usually explore morphological variations at different developmental stages (McDiarmid & Altig, 1999; Provete et al., 2012), with less use of integrative approaches (*e.g*., Köhler et al., 2023). Although it is a consensus that as more evidence added (*e.g*., morphological characters, molecular and ecological data), stronger the species identification (Streicher, Sadler & Loader, 2020; Köhler et al., 2023). This knowledge gap is partially explained by the difficulty in recognizing morphological characteristics of tadpoles, use of features that are difficult to observe (*e.g*., denticles, nostril shape, lateral line, papillae, and spiracle), lack of terminology standardization, and deficiency of identification keys that represent the diversity of species (Andrade et al., 2007; Dubeux et al., 2020a; Pezzuti et al., 2021).

Taxonomic misidentification, of any organism, can lead to a range of problems that affect other disciplines such as physiology, behavior, ecology, and conservation (Bortolus, 2008). In a recent study Dubeux et al. (2022) carry out the first evaluation of the 16S rRNA gene for tadpoles in the northeastern Brazil, highlighting the need for more assertive identifications. For example, according to the authors, the species initially recognized as *Scinax nebulosus* (Spix, 1824) was discovered to be two distinct species after the molecular results (*S*. *nebulosus* and *S*. *auratus* (Wied-Neuwiedi, 1821)). Thus, the molecular data made the authors go back and examine again the tadpoles, trying to find morphological evidence that distinguished both species. In light of this, recent studies have combined molecular tools with morphological data to identify tadpoles more reliably (*e.g*., Randrianiaina et al., 2012; Schulze, Jansen & Köhler, 2015; Amaral et al., 2019; Dubeux et al., 2022). The identification of species from gene sequences when the morphology is unknown, or the morphology is known but it is not enough to distinguish between close related species, is called “reverse taxonomy” (Markmann & Tautz, 2005). In this context, a similar investigation can be applied to amphibians, *i.e*., the tadpole is first recognized as a distinct taxonomic entity and then the adult (*e.g*., Randrianiaina et al., 2011) and could be a powerful tool when applied to species from remote regions (*i.e*., far from major research centers and with a shortage of taxonomists), where there is usually an extensive lack of information on biodiversity (Haddad, Andrade & Cardoso, 1988).

The mid-north sub region of Brazil encompasses the state of Maranhão and half of the state of Piauí, northeastern Brazil. It extends around 42 to 46 W and from 6 S to the Atlantic Ocean in the north (May et al., 1985). The region has a complex landscape due to the contact zone between the ecoregions of the Maranhão Babaçu Forest and Cerrado (Dinerstein et al., 2017), the latter is a biodiversity hotspot (Myers et al., 2000; Mittermeier et al., 2005). The mid-north region of Brazil has unique phytogeographic and climatological characteristics, which results in rich biodiversity and endemism (Martins & Oliveira, 2011) and gives it the status of priority area for conservation (Diniz-Filho et al., 2005). Although this sub region is considered a biodiversity-rich area, there is still an extensive gap in knowledge about the composition and distribution of many groups of organisms, such as amphibians (Martins & Oliveira, 2011; Andrade, Weber & Leite, 2017; Freitas et al., 2017). To date, our understanding of the amphibians of mid-northern Brazil is restricted to a few species’ lists (*e.g*., Bezerra et al., 2012; Andrade, Weber & Leite, 2017; Freitas et al., 2017; França et al., 2021) and the use of some samples in more comprehensive taxonomic studies of some taxa (*e.g*., Gehara et al., 2014). Tadpole research has addressed the hatching and metamorphosis of *Pithecopus hypochondrialis* tadpoles (Matos, Andrade & Hass, 2000) and insecticide toxicity and genotoxicity on the development of *Physalaemus cuvieri* tadpoles (Silva et al., 2013).

Here we present the larval diversity of anuran amphibians from five municipalities located in the eastern Maranhão, mid-northern sub region of Brazil. We used morphological and molecular data to assess the species diversity of tadpoles based on (i) morphological identification based on external characters, (ii) molecular identification from 16S rRNA gene fragment, and (iii) ecological aspects of habitats occupied by each species in the state of Maranhão.

## Materials and Methods

### Study area

**Figure 1:**
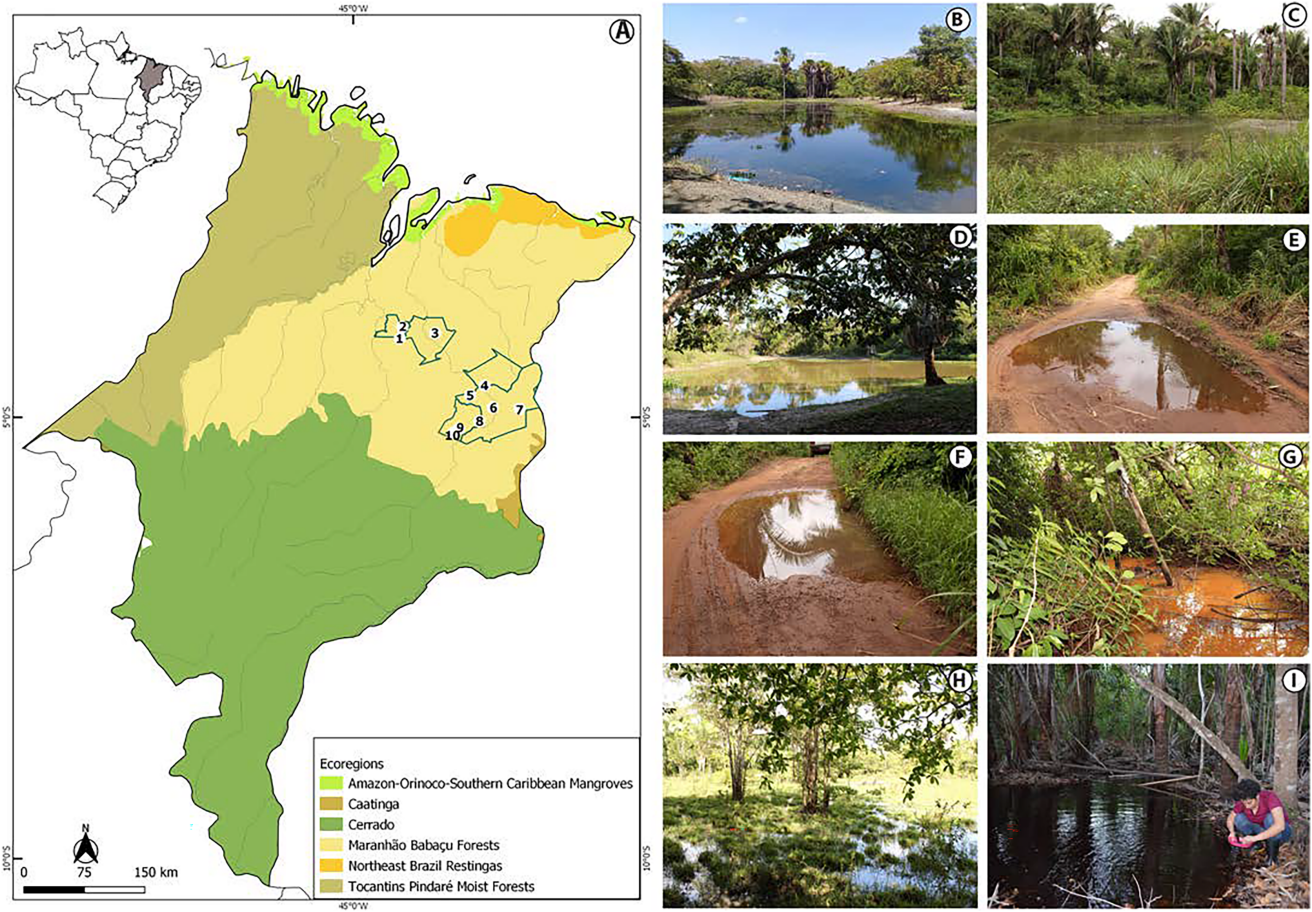
View of the study area in the eastern Maranhão, mid-north region of Brazil. (A) Map of Maranhão highlighting the location of the sampling areas in the following municipalities: São Mateus do Maranhão (1 and 2), Coroatá (3), Aldeias Altas (4), Caxias (5, 6, 7, 8), and São João do Sóter (9, 10). Sampled habitats: (B) perennial ponds; (C and D) dam; (E and F) seasonal ponds; (G) temporary stream; (H) flooded area; (I) perennial pond being sampled by PSS using a 2 mm net. Municipal and ecoregions limits follow (Dinerstein et al., 2017; IBGE, 2019).

The study was conducted in the state of Maranhão, mid-north sub region of Brazil (Fig. 1, Appendix S1). Its geographic space concentrates one of the most complex sets of natural landscapes in the country with the presence of elements from three Brazilian biomes and five ecoregions (Dinerstein et al., 2017; IBGE, 2019) which turns the state into a large transitional or ecotonal area for several groups of organisms (*e.g*., Cavalcanti-Pinto et al., 2019; França et al., 2021; Vieira Serra & Bezerra Almeida, 2021). We sampled in five municipalities from the eastern Maranhão (Fig. 1, Appendix S1) located within the Maranhão Babaçu Forest ecoregion (Fig. 1A) (Dinerstein et al., 2017) and the Cerrado biome (Appendix S2) (IBGE, 2019). In general, the study area exhibits ecotonal characteristics with elements from the Maranhão Babaçu Forest and Cerrado. The climate is characterized by mean annual temperature ranging from 19.2 to 35.7 C (mean = 27.6 ± 5.4 (SD) C) and mean annual precipitation ranging from 5 to 347 mm (mean = 127.2 ± 100.4 mm) (extracted from Fick & Hijmans, 2017). The eastern Maranhão comprises the Parnaíba, Munim, Itapecuru, and Preguiças river basins (Fig. 1).

### Tadpole sampling

We performed 30 samplings along 13 months (from February 2019 to February 2020). Most of the sampling points presented in Appendix S1 were sampled only once, except points 6, 9 and 10 that were sampled twice each. Tadpoles were collected during the day and night in five kinds of habitats: puddles, seasonal and perennial ponds (including flooded areas), dams, and streams (Figs. 1B–1I). We searched for tadpoles by making successive sweeps at various depths of water bodies using a 2-mm mesh sieve or an aquatic entomological net with a 1-mm mesh (known as D-rapiché). The tadpoles were placed in plastic containers containing water from their site of origin and transported to our laboratory. Thus, the tadpoles between stages 34 and 40 (Gosner, 1960) were euthanized by immersion in anesthetic aqueous solution of benzocaine hydrochloride (5g/l) considering minimum animal suffering following the guidelines provided by the Herpetological Animal Care and Use Committee (HACC) of the American Society of Ichthyologists and Herpetologists (Beaupre et al., 2004) and current national legislation (Law no. 11.794/2008). Tadpoles of seven species (*Leptodactylus vastus*, *Osteocephalus taurinus*, *Dendropsophus soaresi*, *Boana multifasciata*, *Leptodactylus pustulatus*, *Physalaemus cuvieri* and *P*. *nattereri*) at stages less than 34 were kept at room temperature in 400 × 180 × 300 mm aquaria oxygenated by air compressors and fed commercial fish food (*e.g*., Alcon^®^ and Nutra Fish^®^). The aquaria were inspected daily until the tadpoles reached developmental stages suitable for morphological identification (*i.e*., ≥stage 34; Gosner, 1960). Development of tadpoles from eggs or early stages in captivity are a common practice in tadpoles’ studies (*e.g*., Alves, Gomes & Silva, 2004; Dubeux et al., 2020a). Although it is known some phenotypic plasticity of tadpoles influenced by the pond characteristics (*e.g*., Relyea & Werner, 2000; Lopes et al., 2020), to date no changes were reported in the oral morphology or general shape of captivity reared tadpoles (Alves, Gomes & Silva, 2004).

We collected at least five fin muscle tissue samples from the anterior portion of the dorsal fin of 181 specimens for molecular analyses of all morphotypes. Next, the tadpoles were fixed and preserved in 5% formalin. The tadpoles (*i.e*., testimony material) are deposited in the Museu da Diversidade Biológica (MDBio) of Universidade Estadual de Campinas (UNICAMP), Campinas, São Paulo (Appendix S3) and the tissue samples are deposited in the collection of the Laboratory of Genetics and Molecular Biology of the State University of Maranhão and Guedes Lab at the State University of Campinas. The sequences were deposited at the GenBank (Appendix S4).

### Ethics statement

The specimens were collected under the approved authorization (71371-3, 31119-2) conceived by Instituto Chico Mendes de Conservação da Biodiversidade (ICMBio/SISBIO) from the Ministério do Meio Ambiente (MMA) of Brazil. The collecting, hold and storage were previously revised by the Ethics and Animal Welfare Committee from Universidade Estadual do Maranhão (002-2021-CEEA/UEMA).

### Morphological identification of tadpoles

We opted to use tadpoles ranging between stages 34 and 40 for identification. We followed several tadpoles’ descriptions and identification keys (*e.g*., Dubeux et al., 2020a, Pezzuti et al., 2021) that say characteristics of the tadpole body can be better observed from 34 stage onwards, as separated toes and the callus on the paws starting to emerge (see Gosner, 1960), that are relevant for robust identification. We identified the tadpoles based on diagnostic characters of external morphology observed under a stereoscopic microscope. For this, we used several taxonomic keys (*e.g*., Rossa-Feres & Nomura, 2006; Pezzuti, Leite & Garcia, 2019; Dubeux et al., 2020a) and we also compared the specimens of tadpoles with original description available for some species (*e.g*., De Sá, 1996; Mercês, Acuña Juncá & Cousiño Casal, 2009; Dubeux et al., 2020b). Morphological measurements were performed as described by Altig & McDiarmid (1999) and Haas & Das (2011) using an AxioCam ICc1 digital camera (Zeiss) coupled to a SteREO Discovery V.8, Carl (Zeiss) stereomicroscope. Morphological nomenclature followed Altig & Johnston (1989), Altig & McDiarmid (1999) and Haas & Das (2011), and oral disc characteristics followed Altig (1970), and Dubois (1995). The identification of developmental stages followed Gosner (1960). We identified 848 specimens morphologically and provided morphological measurements for 355 of these specimens. The color pattern was also taken in consideration of taxonomical identification, this was done based on photographs of recently euthanized individuals, which maintain the characteristics of coloration pattern in life. We edited the images using Adobe Photoshop version 22.4.2 software and assembled the plates using Adobe Illustrator version 25.3.1. Taxonomic nomenclature follows Frost (2023).

### Molecular identification of tadpoles

We extracted total DNA from tadpoles’ fin muscle tissue (except for four species: *Pseudopaludicola* sp., *Boana raniceps*, *Leptodactylus vastus* and *Dendropsophus* cf. *nanus*) using the Wizard^®^ Genomic DNA Purification Kit (Promega, Madison, WI, USA) following the manufacturer’s protocols. Isolation and amplification of genomic regions were performed *via* polymerase chain reaction (PCR) using specific primers 16S rRNA (Palumbi et al., 1991): 16S-L1987 (5′-GCC TCG CCT GTT TAC CAA AAA C-3′) and 16S-H2609 (5′-CCG GTC TGA ACT CAGATC ACG T-3′). The PCR products were purified using ExoProStar 1-Step (GE Healthcare) enzymes following the manufacturer’s protocols. The sequencing reactions were performed using BigDye™ Terminator 3.1 kit (Applied Biosystems, Waltham, MA, USA), and the sequencing products were processed on an ABI 3500/Life Technologies automated capillary system (Applied Biosystems). The PCR had a final volume of 25 µl, composed of 4 µl of 1.25 M DNTPs; 2.5 µl of 10X buffer; 0.5 µl of 25 Mm MgCl_2_; 0.25 µl of forward and reverse primers at 200 nM; 1 µl of DNA sample at 50 ng and 0.2 µl of TAC polymerase at 0.2 U/µl.

To compile a reference library, we accessed the sequences available on the GenBank, identified by their accession number (Appendix S5). *Ceuthomantis smaragdinus* and *Haddadus binotatus* were used as outgroups, following recent published phylogenies (Duellman, Marion & Hedges, 2016; Jetz & Pyron, 2018).

All sequences were aligned in BioEdit (Hall, 1999) using the ClustalW algorithm (Thompson, Higgins & Gibson, 1994). The haplotypes were estimated using the DnaSP 4.10 software (Librado & Rozas, 2009). The most likely substitution model indicated was TN93+G (Tamura & Nei, 1993). The Maximum likelihood (ML) phylogenetic tree was calculated using GTR+G algorithm on MegaX (Kumar et al., 2018). The significance of the clusters was estimated by bootstrap analysis with 1,000 replicates (Felsenstein, 1985). We used a 3% threshold of genetic distances between tadpoles as indicative of new lineages (following Jansen et al., 2011; Amaral et al., 2019). We indicated the bootstrap values of ML in the nodes of the phylogenetic tree.

### Characterization of tadpole’s habitats

The freshwater puddles, seasonal and perennial ponds (including flooded areas), dams, and streams are the habitat of the tadpoles, *i.e*., the environment where the species live (Ricklefs & Relyea, 2013). We provide basic information about the habitat of each species of tadpoles we collected for each waterbody: length, width and depth (both measured with a 50-m tape measure), and general aspect of the vegetation surrounding the water bodies.

## Results

**Figure 2:**
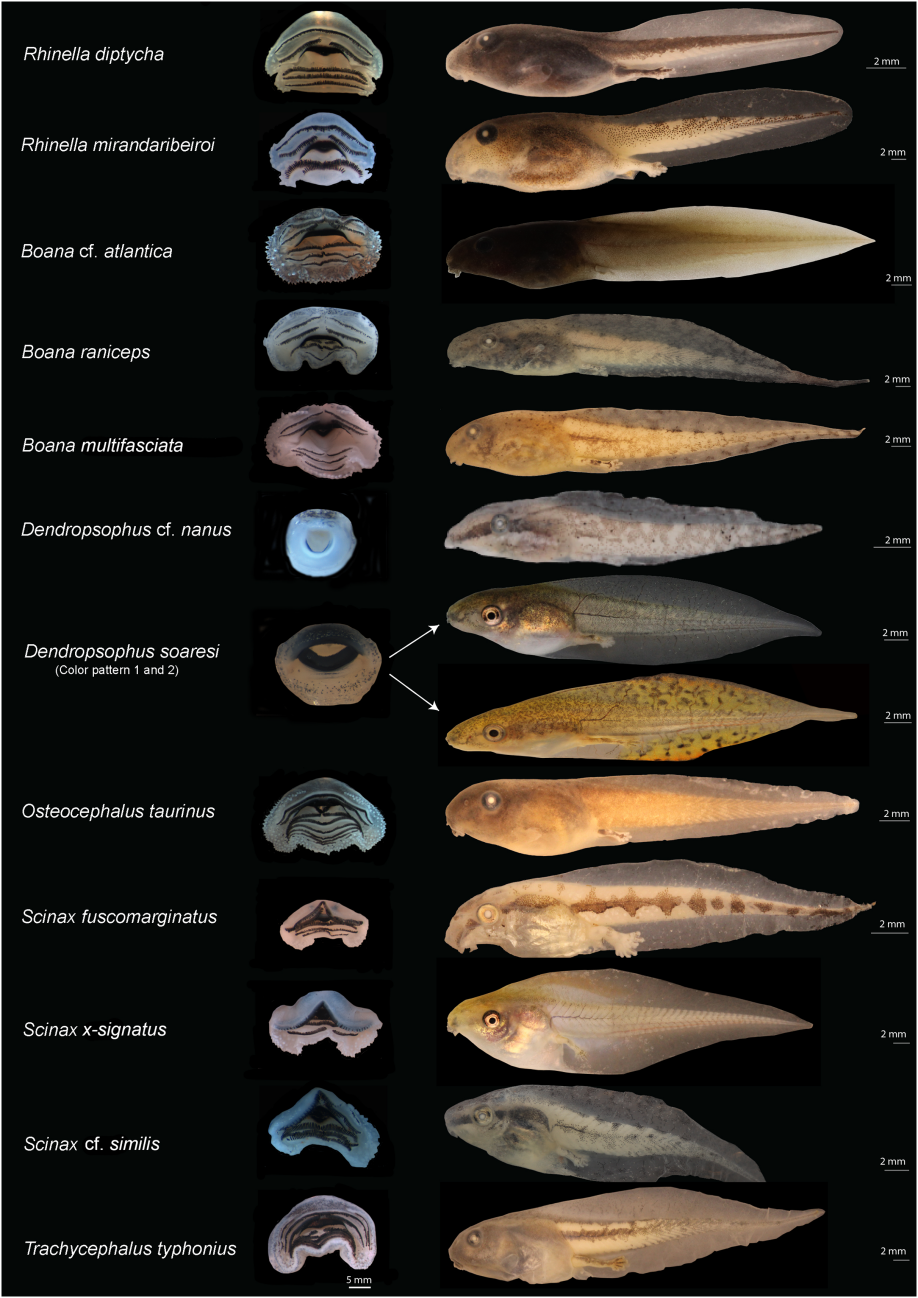
Oral disks (right) and lateral view (left) of the tadpoles of eastern Maranhão, mid-north region of Brazil. *Rhinella diptycha* (TL: 18.9 mm; GS: 37), *Rhinella mirandaribeiroi* (TL: 14.4 mm; GS: 36), *Boana* cf. *atlantica* (TL: 40.1 mm; GS: 37), *Boana raniceps* (TL: 53.7 mm; GS: 36), *Boana multifasciata* (TL: 36.4 mm; GS: 36), *Dendropsophus* cf. *nanus* (TL: 18.9 mm; GS: 37), *Dendropsophus soaresi* (TL: 27.8 mm; GS: 36), *Osteocephalus taurinus* (TL: 27.5 mm; GS: 36), *Scinax fuscomarginatus* (TL: 21.3 mm; GS: 36), *Scinax* x-*signatus* (TL: 36.4 mm; GS: 39), *Scinax* cf. *similis* (TL: 23 mm; GS: 36), *Trachycephalus typhonius* (TL: 39.3 mm; GS: 37). TL: Total length; GS: Gosner stage.

**Figure 3:**
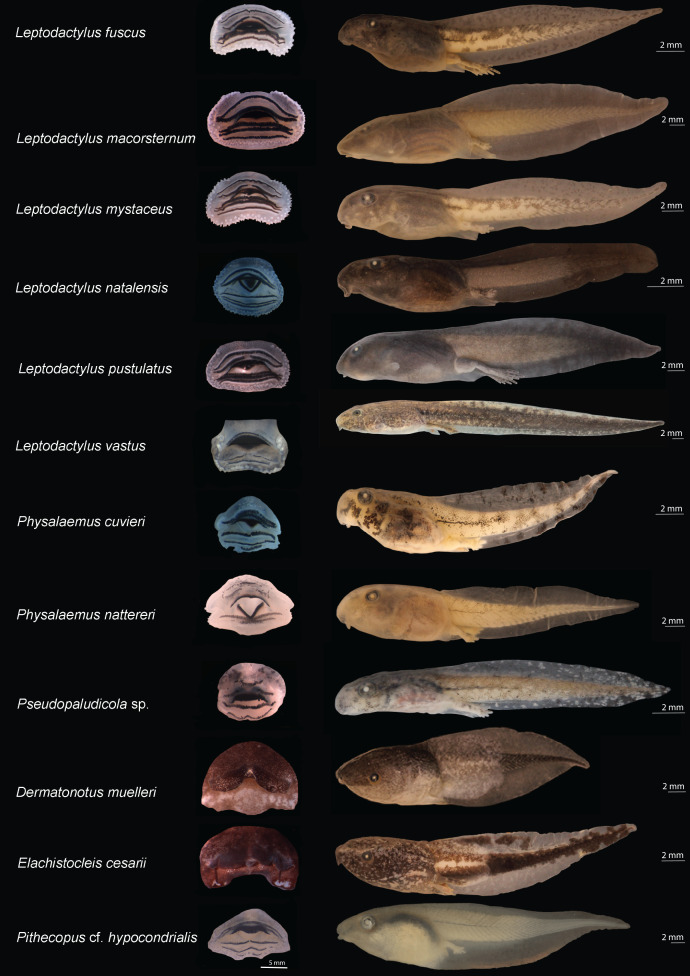
Oral disks (right) and lateral view (left) of the tadpoles of eastern Maranhão, mid-north region of Brazil. *Leptodactylus fuscus* (TL: 31.3 mm; GS: 37), *Leptodactylus macrosternum* (TL: 38.5 mm; GS: 35), *Leptodactylus mystaceus* (TL: 30.6 mm; GS: 36), *Leptodactylus natalensis* (TL: 17 mm; GS: 35), *Leptodactylus pustulatus* (TL: 37.5 mm; GS: 39), *Leptodactylus vastus* (TL: 56.8 mm; GS: 38), *Physalaemus cuvieri* (TL: 19.4 mm; GS: 36), *Physalaemus nattereri* (TL: 29 mm; GS: 37), *Pseudopaludicola* sp. (TL: 19 mm; GS: 36), *Dermatonotus muelleri* (TL: 29.2mm; GS: 36), *Elachistocleis cesarii* (TL: 25.6mm; GS: 35), *Pithecopus* aff. *hypochondrialis* (TL: 39.5 mm; GS: 37). TL: Total length; GS: Gosner stage.

The tadpoles collected in eastern Maranhão represent 26 species belonging to five families (Table 1). The external morphology enabled the identification of 24 species, while the molecular data recognized 22 unique evolutionary units. The most represented family was Hylidae (Hylinae 11 spp., Phyllomedusinae one spp.) followed by Leptodactylidae (Leptodactylinae seven spp., Leiuperinae three spp.), Microhylidae (Gastrophryninae two spp.), and Bufonidae (two spp.) (Tables 1 and 2; Figs. 2 and 3). The tadpoles comprise 12 genera in which the genus *Leptodactylus* (family Leptodactylidae) is the richest containing seven species (Table 1).

**Table 1:** List of tadpole species recorded in the eastern Maranhão, mid-north region of Brazil.

Species	Sampled localities	N
**Bufonidae**		
*Rhinella diptycha* (Cope, 1862)	4, 10	176
*Rhinella mirandaribeiroi* (Gallardo, 1965)	10	84
**Hylidae**		
*Boana* cf. *atlantica* (Caramaschi & Velosa, 1996)	4, 6	25
*Boana multifasciata* (Günther, 1859)	6, 7	25
*Boana raniceps* (Cope, 1862)	8	3
*Dendropsophus* cf. *nanus* (Boulenger, 1889)	8	3
*Dendropsophus soaresi* (Caramaschi & Jim, 1983)	3, 8, 10	192
*Osteocephalus taurinus* Steindachner, 1862	6	80
*Pithecopus* aff. *hypochondrialis* (Daudin, 1800)	10	106
*Scinax* cf. *nebulosus* (Spix, 1824)	1	2
*Scinax* cf. *similis* (Cochran, 1952)	10	55
*Scinax fuscomarginatus* (A. Lutz, 1925)	2	11
*Scinax x-signatus* (Spix, 1824)	2, 3, 5, 10	58
*Trachycephalus typhonius* (Linnaeus, 1758)	10	55
**Leptodactylidae**		
*Leptodactylus fuscus* (Schneider, 1799)	1, 3, 10	172
*Leptodactylus macrosternum* Miranda-Ribeiro, 1926	10	64
*Leptodactylus mystaceus* (Spix, 1824)	10	91
*Leptodactylus natalensis* Lutz, 1930	7, 9	97
*Leptodactylus pustulatus* (Peters, 1870)	4	43
*Leptodactylus troglodytes* Lutz, 1926	10	6
*Leptodactylus vastus* Lutz, 1930	3, 10	41
*Physalaemus cuvieri* Fitzinger, 1826	3, 8, 10	85
*Physalaemus nattereri* (Steindachner, 1863)	10	81
*Pseudopaludicola* sp.	4	2
**Microhylidae**		
*Dermatonotus muelleri* (Boettger, 1885)	10	46
*Elachistocleis cesarii* (Miranda-Ribeiro, 1920)	3	6

**Note:**

Sampled localities = municipalities of São Mateus do Maranhão (1 and 2), Coroatá (3), Aldeias Altas (4), Caxias (5, 6, 7, 8) and São João do Sóter (9, 10).

**Table 2:** Characteristics parameters of external morphology of the tadpoles recorded to the eastern Maranhão, mid-north region of Brazil.

Species	Gosner stage	Jaw sheath shape + LKRF	Oral disk emargination
**Bufonidae**			
*Rhinella diptycha* (*N* =20)	34, 35, 36, 37, 38	arc-V, 2(2)/3, 2(2)/3(1), 2(2)/2	Lateral
*Rinella mirandaribeiroi* (*N* = 41)	34, 35, 36, 37, 39, 40	arc-V, 2(2)/3(1)	Lateral
**Hylidae**			
*Boana* cf. *atlantica* (*N* = 9)	34, 35, 36, 37	arc-V, 2(1,2)/3(1)	Ventral
*Boana raniceps* (*N* = 2)	36, 39	arc-V, 2(1,2)/3(1)	Ventral
*Boana multifasciata* (*N* = 13)	35, 36, 37, 38, 39	M-V, 2(1,2)/3(1)	Ventral
*Dendropsophus *cf.* nanus* (*N* = 1)	37	arc-V, 0/0	_
*Dendropsophus soaresi* (*N* = 15)	35, 36, 37	arc-U, 0/1	_
*Osteocephalus taurinus* (*N* = 19)	34, 35, 36, 37, 39, 40	arc-V, 2(2)/6(1), 2(2)/7(1)	Ventral
*Pithecopus* aff. *hypochondrialis* (*N* = 20)	36-37-38	M-V, 2(2)/3(1)	_
*Scinax fuscomarginatus* (*N* = 2)	34, 36	M-V, 2(2)/3	Ventral
*Scinax x-signatus* (*N* = 8)	34, 35, 39, 40	M-V, 2(2)/3(1)	Ventral
*Scinax* cf. *similis* (*N* = 21)	34, 35, 36, 3, 38, 39	M-V, 2(1,2)/3(1)	Ventral
*Trachycephalus typhonius* (*N* = 22)	34, 35, 36, 37, 38, 40	arc-V, 3(1,3)/5(1), 3(1,3)/5(1,2), 3(1,3)/5(1,2,3,4)	Ventral
			
**Leptodactylidae**			
*Leptodactylus fuscus* (*N* = 20)	35, 36, 37	arc-V, 2(2)/3(1)	_
*Leptodactylus macrosternum* (*N* = 21)	34, 35, 36	arc-V, 2/3	_
*Leptodactylus mystaceus* (*N* = 20)	35, 36, 37, 38	arc-V, 2(2)/3(1)	_
*Leptodactylus natalensis* (*N* = 20)	34, 35	arc-V, 2/3	_
*Leptodactylus pustulatus* (*N* = 6)	38, 39, 40	arc-V, 2(2)/3	_
*Leptodactylus vastus* (*N* = 14)	35, 36, 38	U-V, 1/ 2(1), 1/ 2	_
*Physalaemus cuvieri* (*N* = 20)	34, 35, 36, 37, 38, 39	arc-V, 2(2)/3(1)	Lateral/ventral
*Physalaemus nattereri* (*N* = 20)	34-35-36-37-38	M-V, 2(2)/3(1)	Lateral
*Pseudopaludicola* sp. (*N* = 1)	36	arc-V, 2(2)/2	Lateral
**Microhylidae**			
*Dermatonotus muelleri* (*N* = 15)	34-35-36-37	_	_
*Elachistocleis cesarii* (*N* = 5)	34-35-36	_	_

**Note:**

Gosner stage presented only for tadpoles between 34 and 40 stages; Jaw sheath shape is given first for the upper, then for the lower part. LKRF, labial keratodont row formula presented by anterior (A-) and posterior (P-) rows, gaps are inside brackets and backslash separate the upper and lower jaw sheath

The tadpoles presented herein were in three puddles, 21 seasonal and two perennial ponds (including flooded areas), eight dams, and 11 streams. These water bodies were surrounded by cerrado and babaçu forest vegetation (Table 3).

**Table 3:** Characteristics of the habitats of the tadpoles recorded in the eastern Maranhão, mid-north region of Brazil.

Sampled locations	Length	Width	Depth	Vegetation
Point 1*	10	6	10	Cerrado
Point 2*	>50	>50	30	Cerrado
Point 3	9	1	3	Cerrado
Point 4	>50	>50	80	Cerrado
Point 5	>50	>50	50	Babaçu forest
Point 6	>50	>50	5	Cerrado
Point 7	14	5	3	Babaçu forest
Point 8	13	6	4	Babaçu forest
Point 9	>50	>50	6	Babaçu forest
Point 10	3	1	8	Babaçu forest

**Notes:**

Length, puddle length (m); Width, puddle width (m); Depth, depth of the puddle (cm); Vegetation: classification of the local vegetation around the water bodies.

Detailed about the sampled points are provided in Fig. 1 and Appendix S1.

* Habitats located nearby anthropic environments, including some puddles at the edges of access roads.

### Morphological identification

We identified 24 species morphologically (Table 2; Appendix S6). The color pattern and morphometric characteristics showed greater inter and intraspecific variation, thus contributing to a lesser extent to the identification of tadpoles when compared to oral morphology (Figs. 2 and 3; Table 2; Appendix S6). An overview of the characters we recognized for each species is detailed below.

*Rhinella diptycha* showed jaw sheath shape arc-V and labial keratodont row formula 2(2)/3, 2(2)/3(1), 2(2)/2 (Table 2), and *Rhinella mirandaribeiroi* presented a globular body (Fig. 2).

Bufonid tadpoles of both *R. diptycha* and *R. mirandaribeiroi* had a shorter total length when compared to what has already been known (17.9 mm in *R*. *diptycha* and 15.4–15.7 mm in *R*. *mirandaribeiroi*; Appendix S6).

Among hylids, we provide for the first time morphometrical data for *Boana multifasciata* tadpole (see Appendix S6) that showed total length 36.9–40.3 mm; we also provide the first images of the oral disc of this tadpole (Fig. 2), along with information on characters such as the shape of the nostrils reniforms, snout oval in dorsal view and rounded in lateral view, and jaw sheath shape and labial keratodon line formula M-V, 2(1,2)/3(1) (Table 2). *Boana* cf. *atlantica* showed jaw sheaths shape arc-V and labial keratodont row formula 2(1,2)/3(1), 2(1,2)/3(1). *Osteocephalus taurinus* tadpoles presented an interruption in the first row of denticles in the lower part of the oral apparatus and one row of denticles less, jaw sheath shape and labial keratodont row formula is arc-V 2(2)/6(1), 2(2)/7(1). *Trachycephalus typhonius* exhibited arc-V labial keratodont row formula 3(1,3)/5(1), 3(1,3)/5(1,2), 3(1,3)/5(1,2,3,4) (Table 2). *Dendropsophus soaresi* exhibited coloration in life consisting of olive green to light brown with golden dots most evident in the flank region (Fig. 2). The tail muscle was finely reticulated by olive green to light brown melanophores but translucent in its larger portion. The fins were finely reticulated and translucent, but some specimens showed diffusely scattered golden dots on the fins. The venter was translucent up to the eye region, where it turned silvery white. We also observed variation in body, tail, and fin coloration between the different habitats in which the tadpoles were collected; lighter colors (yellow fins with black melanophores) were observed in muddy water sites (seasonal ponds), and darker colors in more turbid waters (perennial ponds) (Fig. 2).

We also reported new characters we recognized in leptodactylid tadpoles (Fig. 3). For example, *Leptodactylus fuscus* showed a sinistral spiracle, positioned lateroventrally, directed posterodorsally, with a centripetal wall with a small free end and an inner wall larger than the outer wall. *Leptodactylus natalensis* showed oral disc positioned anteroventrally, without emarginations; the body ranged from black to dark brown, with dark spots uniformly distributed; fins were translucent, with spots finely reticulated by melanophores; upper portion of the tail muscle was darker than the lower portion, finely reticulated by melanophores; venter was translucent and finely reticulated by melanophores.

In microhylids (Fig. 3), *Dermatonotus muelleri* showed a spiracle centripetal wall slightly larger than the inner wall; coloration in life was light brown to reddish-brown, with white spots on the posterior portion of the body extending to the middle third of the tail, where the muscle becomes darker and the fins more translucent; tadpoles collected in muddy puddles showed lighter color, whereas those collected in turbid water were darker; the venter was silvery-white in some specimens and opaque white in others; the center of the venter is predominantly white, and the sides contain dark brown clusters. *Elachistocleis cesarii* showed dark brown coloration; body with numerous white/cream dots extending to the venter; tail muscle with a white line extending from the body-tail junction to the anterior third of the tail; fins mostly translucent, diffusely pigmented with the same color as the body; and venter more densely pigmented to the eye region, where it becomes more diffusely colored with white/cream and light brown spots. The dermal flaps varied individually; one specimen had a short, slightly undulated flap and the others had large, undulated flaps.

*Leptodactylus vastus*, *Pseudopaludicula* sp., *Dendropsophus* cf. *nanus*, and *Boana raniceps* were identified based only on external morphology. *Leptodactylus vastus* reached the imago stage, corroborating the identification of the tadpole.

Unfortunately, we could not include the species *Pseudopaludicula* sp., *Dendropsophus* cf. *nanus*, and *Boana raniceps* in molecular studies this time. Thus, they were identified based solely on morphological characters. *Pseudopaludicola* sp. showed morphological and morphometric similarities with the tadpole of *Pseudopaludicola mystacalis*, however since we had a limited sample (one specimen above stage 34), we decided to maintain the designation as *Pseudopaludicola* sp. to avoid making an identification error. A similar case happened with the species *Dendropsophus* cf. *nanus* that showed morphological and morphometric similarities with the tadpole of *Dendropsophus nanus*.

### Molecular identification

We analyzed 126 sequences of a 556-bp fragment of the mitochondrial 16S rRNA gene and obtained 324 conserved, 230 varied, and 220 informative sites for parsimony and 57 haplotypes. These sequences represent 11 genera and 22 species. Our reference library was composed of 22 sequences from GenBank (Appendixs S4 and S5). The genetic divergence matrix was organized by species with averages calculated for intra- and interspecific divergence (Appendix S7). The sequences available for adult amphibians on the BLAST platform corroborated 17 of the 24 taxonomic identifications of tadpole species based on morphology (Appendix S8). Thus, our results indicate that the 16S rRNA specific primer was suitable for molecular identification of tadpoles.

The average interspecific genetic divergence ranged from 8.34 to 27.49%; the lowest distance was recorded between *Osteocephalus taurinus* and *Trachycephalus typhonius*, whereas the highest distance was recorded between *Leptodactylus macrosternum* and *Scinax fuscomarginatus*. The mean intraspecific divergence ranged from 0.0% in *Boana* cf. *atlantica* to 2.31% in *Elachistocleis cesarii* (Appendix S7).

**Figure 4:**
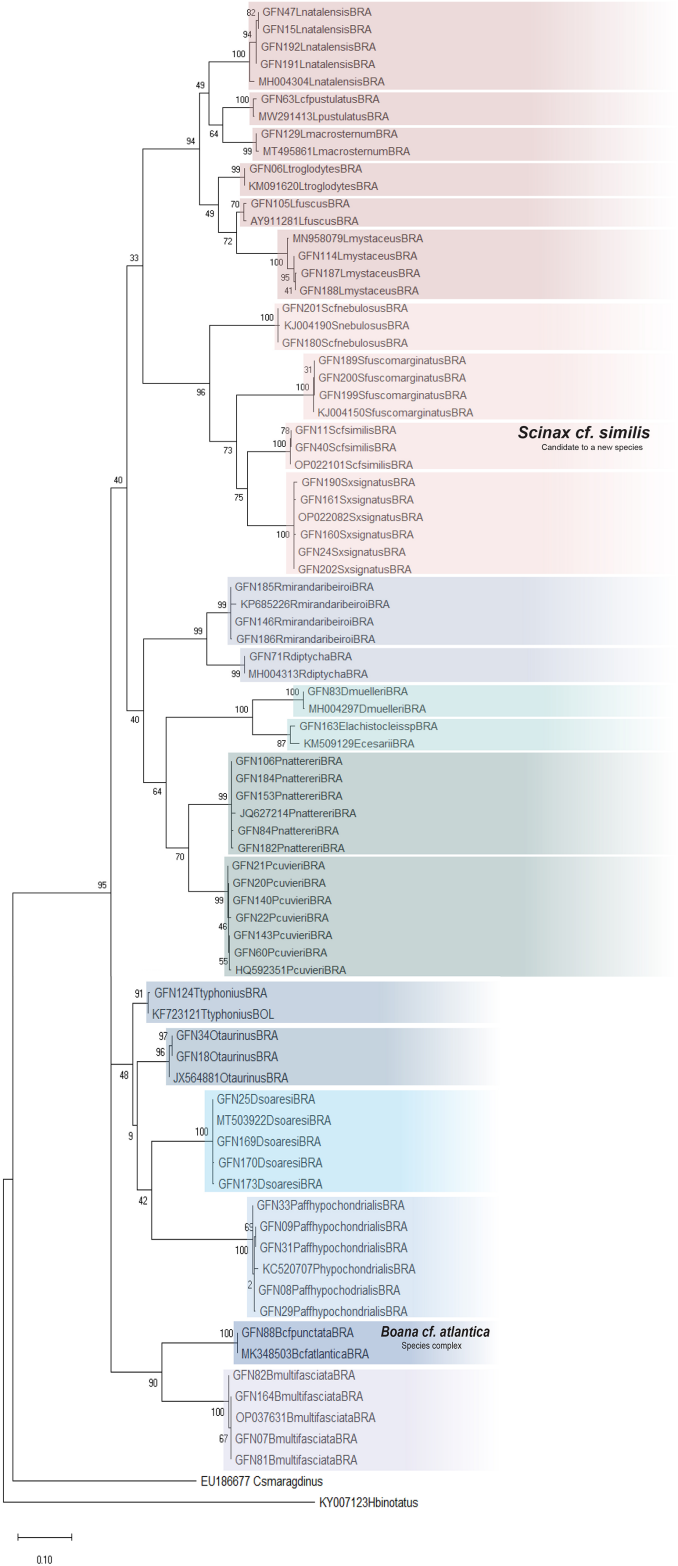
Maximum Likelihood tree of the mitochondrial gene rRNA 16S. Numbers on branches indicate *bootstrap* support Maximum Likelihood (ML). Tadpoles sampled in this study show the acronym GFN (Guedes Field Number) and a number before the species name. All remaining specimens comprise adults from the reference database (GenBank, Appendix S5).

The ML was strongly supported by high bootstrap values (Fig. 4). Regardless of the phylogenetic tree, which included tadpole sequences from this study and adult sequences from GenBank, most tadpole species (*n* = 17) identified morphologically clustered tightly with the corresponding adult species. We adopted the terminology *Scinax* cf. *similis*, *Boana* cf. *atlantica*, and *Pithecopus* aff. *hypochondrialis* respectively, based on molecular sequences we compared (Appendix S4) because there is evidence that these lineages represent species complex.

Although *Leptodactylus troglodytes* and *Scinax* cf. *nebulosus* seems to be common species in eastern Maranhão (TB Guedes, 2019, personal observations), we obtained data to conduct only molecular identification. These species were collected only once at developmental stages ≤28 (Gosner, 1960), even though multiple expeditions were performed.

This study also provides new records of occurrences for the state of Maranhão. We present the first record of *Leptodactylus natalensis* for the interior of the state of Maranhão, 385 km away from Ilhas Canários, Delta do Parnaíba municipality (Leite et al., 2008); this species was previously known only from the coastal region of Brazil (Frost, 2023). We also provide the first record of *Leptodactylus pustulatus* in Maranhão, in Aldeias Altas municipality, located 289 km away from Sete Cidades National Park, state of Piauí (Araújo et al., 2020); this species is known to occur in arid and semiarid regions of northeastern Brazil (Frost, 2023). Our finding of *Elachistocleis cesarii* is also the first record for the state of Maranhão.

We also extend the distribution of some species already known to occur in Maranhão. Our record of *Scinax fuscomarginatus* extends its distribution 212 km (in a straight line) north from the Mirador State Park in Maranhão, the geographically nearest record (Andrade, Weber & Leite, 2017). *Leptodactylus macrosternum* present wide distribution (Magalhães et al., 2020); however, we collected the tadpoles for the first time in the municipality of São João do Soter, 355 km from Floriano (Piauí), the nearest location where the species have been recorded (Santos et al., 2014). *Pithecopus* aff. *hypochondrialis* was recorded in São João do Soter, 336 km west of the Gurupi Biological Reserve (Freitas et al., 2017); this is the second record of occurrence of the species in the Maranhão.

### Habitat characteristics

Tadpoles were collected in water bodies of various dimensions, ranging from 3 to 50 m long, 1 to 50 m wide, and 3 to 80 cm depth (Table 3). The vegetation around the water bodies varied between typical babaçu forest vegetation and cerrado. Beyond mention the surrounded vegetation of the water bodies, it is also worth to mention the habitats sampled in the municipality of São Mateus do Maranhão (points 1 and 2; Appendix S1) were located nearby anthropic environments, including some puddles at the edges of access roads (Table 3).

The tadpoles of *R*. *mirandaribeiroi*, *B*. *raniceps*, *D*. cf. *nanus*, *P. cuvieri*, *P. nattereri*, and *L. natalensis* were collected in puddles up to 30 cm depth with little cerrado vegetation at the edges. *Dendropsophus soaresi*, *Scinax x-signatus*, *T*. *typhonius*, *L*. *macrosternum*, and *D*. *muelleri* were collected in puddles and dams 50–80 cm depth with shrubby babaçu forest vegetation on the banks. *Pithecopus* aff. *hypochondrialis*, *L*. *fuscus*, and *L*. *vastus* were habitat generalists, found in all kinds of waterbodies with cerrado and babaçu forest shrubs vegetation at the edges. *Elachistocleis cesarii* was collected in a 50 cm depth puddle, with little cerrado vegetation on the bank but we observed submerged vegetation.

The tadpoles of *Boana multifasciata*, *B*. cf. *atlantica*, *O*. *taurinus*, and *L*. *mystaceus* were collected in different streams about 20 cm depth, with dense cerrado vegetation on the banks and large amounts of organic matter at the bottom. *Leptodactylus pustulatus* and *Pseudopaludicula* sp. were collected on the bank of a stream (5 cm depth) during the dry season. *Scinax x-signatus* was found in a shallow puddle (5 cm depth) with nearby bushes. *Rhinella diptycha* was collected at the edge of dams at 10 cm depth, with little mixed cerrado and babaçu forest vegetation at the edges. *Scinax fuscomarginatus* was found in a flooded area with a large amount of vegetation in the water but little mixed cerrado and babaçu forest vegetation at the edges. The waterbodies in which *Rhinella diptycha* and *S*. *fuscomarginatus* were collected did not contained tadpoles of any other species.

## Discussion

Our results show that careful combination of external morphology and genomic data proved to be a powerful tool to determine the diversity of tadpoles from eastern Maranhão, mid-north Brazil. The species diversity of tadpoles we identified (26 spp.) represents 23.1% of the amphibian diversity known based on a recently published report of Maranhão’s biodiversity by Pavan & Moraes (2023). They recorded 112 species of amphibians for Maranhão state (see table 2.3.1 in Pavan & Moraes, 2023) based on morphology of adults; however it seems they also included species sampled in Tocantins and Piauí, states that border Maranhão state (see page 56 in Pavan & Moraes, 2023); among the 18 municipalities they sampled only 10 are inside Maranhão limits using data based on morphology of adults (Pavan & Moraes, 2023); The state of Maranhão shows 217 municipalities. All these led us to believe that we still do not have a reliable or stable number of amphibians species for the Maranhão state to compare.

Among the morphological characters our results showed that oral morphology was one of the most important and conserved characters for identifying tadpoles based on external morphology, and the specific 16S rRNA primer was suitable for molecular identification. Thus, this approach we used provided robust evidence to recognize the evolutionary entities studied here, enabling us to characterize the rich (tough poorly known) biodiversity of eastern Maranhão. The oral morphology stands as one of the most important morphological characters for tadpoles properly identification corroborating the literature (*e.g*., Dubeux et al., 2020a) as well as the use of DNA barcoding for identification of anuran larvae (*e.g*., Schulze, Jansen & Köhler, 2015; Dubeux et al., 2022).

We identified 24 species based on external morphology, and 20 of them were corroborated by molecular identification. Unlike previous studies published for amphibians of Maranhão state (*e.g*., Bezerra et al., 2012; Andrade, Weber & Leite, 2017; Freitas et al., 2017; França et al., 2021; Pavan & Moraes, 2023), this study pioneers the use both morphological and molecular data to identify tadpoles in the state of Maranhão. This approach has been effective in previous studies conducted in diverse natural regions (*e.g*., Randrianiaina et al., 2011, 2012; Schulze, Jansen & Köhler, 2015), and it was also recently used to identify tadpoles in fragments of the Atlantic Forest, in the southeastern and northeastern Brazil (Amaral et al., 2019; Dubeux et al., 2020a, 2022). In Pavan & Moraes (2023) recent list 12 species are presented as *species incerta* (sp.), *confer* (cf.), *species affinis* (aff.), or *ex grege* (gr.) highlighting the importance of genomic data to identify even adult of amphibians’ species in the state.

Careful analysis of external morphological characters and comparison with published literature allowed us to identify 95% of the species reported here. Moreover, this analysis enabled us to recognize morphological variations and observe additional characters never reported for previously described species, which adds taxonomic value to this synthesis that goes beyond a species list. For example, some specimens (*n* = 30) of the monophyletic lineage identified herein as *D*. *soaresi* showed coloration similar to that described for *Dendropsophus minutus*, while other specimens (*n* = 162) showed coloration similar to that of *D*. *melanargyreus* (Schulze, Jansen & Köhler, 2015) (Fig. 2). Another character that varied in size and shape was the dermal flap in *E*. *cesarii* (see details in Appendix S9) (Rossa-Feres & Nomura, 2006). In *R*. *mirandaribeiroi*, body, tail, and total length differed slightly from literature available (Grosso et al., 2020).

The morphological analysis of a large sample of tadpoles from mid-northern Brazil (Table 1) allowed us to identify additional characters to the recorded species, thus complementing the literature (showed in Table 2, Figs. 2 and 3, and Appendix S6). This observation also shows that there is a gap in the knowledge of the morphology of described taxa. We examined 20 specimens of *Rhinella* ranging between stages 34 and 38, and we compared them to the data provided by Mercês, Acuña Juncá & Cousiño Casal (2009). We found some variations especially regarding the total length (17.9 mm in our study *vs*. 20 mm in Mercês, Acuña Juncá & Cousiño Casal, 2009); some variation was also found on LTRF (arc-V, 2(2)/3, 2(2)/3(1), 2(2)/2) for some specimens. Although both studies considered specimens on similar stages we believe these variation could be explained as intraspecific variation. Although we are aware that differences in total length can be found when comparing distinct populations, we did not investigate population variations in this study. For *Boana* cf. *atlantica* + *B*. *punctata*, *O*. *taurinus*, and *P*. aff. *hypochondrialis*, we present novel morphological characters (*e.g*., body shape, jaw sheath shape and labial tooth row formula) that support the hypothesis of the existence of a species complex that needs to be further investigated (Bruschi et al., 2013; Jungfer et al., 2013; Lima et al., 2019). Moreover, we provide basic data for the characterization of *Rhinella mirandaribeiroi* tadpoles collected at a site closer to the type locality (Frost, 2023) and associated environmental parameters. This information increases the knowledge about this species categorized as Data Deficient by the International Union for Conservation of Nature (IUCN, 2021).

For the tadpoles of *Boana multifasciata*, new information on external morphology is presented, morphometric data (Table 2 and Appendix S6), as first information on the habitat of these tadpoles, the description of these new characters provides important diagnostic information for this species. The tadpole originally described by De Sá (1996) presents characters different from those found here in, such as the oval snout in dorsal view and rounded in lateral view, reniform nostrils and labial keratodon line formula 2(1,2)/3(1).

The species *Boana* cf. *atlantica* and *Scinax* cf. *similis* require further investigation. Both species are typical of the Atlantic Forest (Lima et al., 2019; Dubeux et al., 2022), however the closest records are far 1,316 km from our study area.

The tadpole identified by us morphologically as *Pithecopus hypochondrialis*, was identified molecularly as belonging to the *Pithecopus* aff. subclade *hypochondrialis*. In a study that evaluated the taxonomic status of populations attributed to *P*. *hypochondrialis* Bruschi et al. (2013) established the existence of a subclade that involved the populations of three municipalities in Maranhão (São Luís, Bacabeira and Urbano Santos) and respectively a population of Tocantins, Bahia and Minas Gerais. The authors emphasize the need for a careful reanalysis of the phenotypic characteristics used to discriminate *P*. *azurea* and *P*. *hypochondrialis*. Therefore, in this work we chose to use the terminology of *Pithecopus* aff. *hypochondrialis*.

The molecular data *for Dendropsophus soaresi* aligned with previous phylogenies (Orrico et al., 2021); the confirmation of the species allowed us to report morphological information (Table 2, Fig. 2, Appendix S6) from closer to the type locality (Frost, 2023), the first habitat information for the tadpole in Brazilian territory, and also extend the distribution for the species (Andrade, Weber & Leite, 2017).

There is a shared concern among herpetologists (Pavan & Moraes, 2023) and other zoologists (IMESC, 2023) regarding the increasing and constant destruction of natural environments in the state of Maranhão, including fragmentation of natural landscapes that are reduced to, in general, small, isolated and with an intense edge effect. The area of Cerrado is also constant set of fire, areas of natural vegetation are cut down to remove wood or for use as animal pasture. Our tadpole collections were carried out in municipalities that were on the list of those with the most deforestation in the MATOPIBA (Maranhão, Tocantins, Piauí and Bahia states in Brazil) region in the years 2020 to 2021 (http://ipam.org.br) and places known to have suffered vast environmental degradation (Marques et al., 2020). This increase even more the relevance of our study, which can be the testimony of the amphibian diversity of an area under destruction.

## Conclusion

Our study is the first synthesis using both morphological and molecular data to assess the amphibians’ diversity of Maranhão. Despite the short-term sampling (13 months) in a restricted area (ten sampling sites in five municipalities), our study reveals a high number of tadpole (amphibian) species in the region, identifies species that require further taxonomic and systematic attention (species complex), and extends the geographic distribution of six species, three of which represent new occurrences for the state. Our results strengthen the hypothesis that the diversity of amphibians from Maranhão is underestimated. Surveying this diversity require substantial fieldwork effort, sampling in different seasons, and the use of integrative taxonomical approaches. There is an urgent need to assess the status of species/lineages from mid-northern Brazil, as this area is under intense pressure from habitat loss. Our work highlights the importance of herpetological inventories in poorly sampled areas, decentralizing the knowledge of biodiversity from the surroundings of large cities and renowned research centers.

## Supplemental Information

10.7717/peerj.16640/supp-1 Supplemental Information 1 Spatial and climatic data (extracted from WorldClim: Fick & Hijmans, 2017) of the study area in the eastern Maranhão, mid-north region of Brazil. Annual Temp. = annual temperature average (^o^ C); Anual Rain. = annual rainfall average (mm); Season of sampling = rainy season from December to April, dry season from May to November. Most of the sampling points presented in Appendix S1 were sampled only once, except points 6, 9 and 10 that were sampled twice each.

10.7717/peerj.16640/supp-2 Supplemental Information 2 View of the study area in the eastern Maranhão, mid-north region of Brazil. (A) Map of Maranhão highlighting the location of the sampling areas in the following municipalities: São Mateus (1 and 2), Coroatá (3), Aldeias Altas (4), Caxias (5, 6, 7, 8), and São João do Sóter (9, 10). Municipality and biome limits follow (IBGE, 2019).

10.7717/peerj.16640/supp-3 Supplemental Information 3 Voucher numbers for the specimens of the present study (organized by lots considering collection data, locality and species) housed at the Museum of Biological Diversity – Zoology area (MDBio – ZUEC), of the Institute of Biology, State University of Campi.

10.7717/peerj.16640/supp-4 Supplemental Information 4 57 GenBank accession numbers.

10.7717/peerj.16640/supp-5 Supplemental Information 5 22 GenBank accession numbers for the gene rRNA 16S used as reference library in the present study.

10.7717/peerj.16640/supp-6 Supplemental Information 6 Morphometric data of the tadpoles of the eastern Maranhão, mid-north region of Brazil. BL = body length from snout to the point where the axis of the tail myotomes meets the body wall; TAL = tail; TL = total length; ED = eye diameter; IOD = interorbital distance; IND = internarial distance (center to center); ODW = oral disk width; BH = body height; BW = maximum body width; MTH = maximum tail height; TMH = tail muscle height at body-tail junction, where ventral line of musculature meets trunk contour.

10.7717/peerj.16640/supp-7 Supplemental Information 7 Percentage of nucleotide divergences generated for the gene rRNA 16S for the tadpole species in the present study. The numbers on the diagonal, in bold, correspond to the intrapopulation mean.

10.7717/peerj.16640/supp-8 Supplemental Information 8 Comparison between the results from morphological characterization and molecular identification. Molecular identification was performed by comparing the sequences generated in this study and those provided in the BLAST platform.

10.7717/peerj.16640/supp-9 Supplemental Information 9 Variation in the shape of the dermal flap of *Elachistocleis cesarii*, the first photo (up) at stage 35 of Gosner (1960), the second was observed at stages 34 to 36 (down). The short, paired semicircular dermal flaps in front of the mouth in the first photo and long semicircular and significantly exceeding the front of the mouth in the second photograph

## Additional Information and Declarations

### Field Study Permissions

The following information was supplied relating to field study approvals (*i.e*., approving body and any reference numbers): The specimens used in this study were collected under the approved authorization (71371-3, 31119-2) conceived by Instituto Chico Mendes de Conservação da Biodiversidade (ICMBio/SISBIO) from the Ministério do Meio Ambiente (MMA) of Brazil. The collecting, hold and storage were previously revised by the Ethics and Animal Welfare Committee from Universidade Estadual do Maranhão (002-2021-CEEA/UEMA).

### DNA Deposition

The following information was supplied regarding the deposition of DNA sequences: The sequences are available at GenBank and the 79 accession numbers are available in the Supplemental Files.

### Data Availability

The following information was supplied regarding data availability: The data is available in the Supplemental Files.

## References

[ref-1] Altig R (1970). A key to the tadpoles of the continental United States and Canada. Herpetologica.

[ref-2] Altig R, Johnston GF (1989). Guilds of anuran larvae: relationships among developmental modes, morphologies and habitats. Herpetological Monographs.

[ref-3] Altig R, McDiarmid RW, McDiarmid RW, Altig R (1999). Body plan: development and morphology. Tadpoles: The Biology of Anuran Larvae.

[ref-4] Alves AC, Gomes MDR, Silva SP (2004). Description of the tadpole of *Scinax auratus* (Wied-Neuwied) (Anura, Hylidae). Revista Brasileira de Zoologia.

[ref-5] Amaral CRL, Chaves ACS, Borges Júnior VNT, Pereira F, Silva BM, Silva DA (2019). Amphibians on the hotspot: molecular biology and conservation in the South American Atlantic rainforest. PLOS ONE.

[ref-6] Andrade GV, Eterovick PC, Rossa-Feres DC, Schiesari L, Nascimento LB, Oliveira ME (2007). Estudos sobre girinos no Brasil: histórico, conhecimento atual e perspectivas. Herpetologia no Brasil II.

[ref-7] Andrade EB, Weber LN, Leite JRSA (2017). Anurans of the Parque Estadual do Mirador, a remnant of Cerrado in the state of Maranhão, Northeastern Brazil. Biota Neotropica.

[ref-8] Araújo KC, Andrade EB, Brasileiro AC, Benício RA, Sena FP, Silva RA, Santos AJS, Costa CA, Ávila RW (2020). Anurans of Sete Cidades National Park, Piauí state, northeastern Brazil. Biota Neotropica.

[ref-9] Beaupre SJ, Jacobson ER, Lillywhite HB, Zamudio K (2004). Guidelines for use of live amphibians and reptiles in field and laboratory research. Herpetological animal care and use committee (HACC) of the American Society of Ichtyologists and Herpetologists. https://ssarherps.org/wp-content/uploads/2014/07/guidelinesherpsresearch2004.pdf.

[ref-10] Bezerra KC, Oliveira RJF, Conceição E, Pavan D, Fraga EC, Barros MC, Barros MC (2012). Anfíbios da área de Proteção Ambiental Municipal do Inhamum, Caxias/MA. Biodiversidade na Área de Proteção Ambiental Municipal do Inhamum.

[ref-11] Bortolus A (2008). Error cascades in the biological sciences: the unwanted consequences of using bad taxonomy in ecology. AMBIO: A Journal of the Human Environment.

[ref-12] Bruschi DP, Busin CS, Toledo LF, Vasconcellos GA, Strussmann C, Weber LN, Lima AP, Lima JD, Recco-Pimentel SM (2013). Evaluation of the taxonomic status of populations assigned to *Phyllomedusa hypochondrialis* (Anura, Hylidae, Phyllomedusinae) based on molecular, chromosomal, and morphological approach. BMC Genetics.

[ref-13] Cavalcanti-Pinto K, Pinheiro LR, Ferreira JB, Cruz LSS, Anjos SF, Pereira KDL, Araújo DM, Castro LS (2019). Herpetological biodiversity of the Maranhão ecotone, Brazil. Scientific Eletronic Archives.

[ref-14] da Silva CT, Eskinazi-Sant’Anna EM, Silvério Pires MR (2020). Environmental drivers of tadpole community structure in temporary and permanent ponds. Limnologica.

[ref-16] De Sá RO (1996). Hyla multifasciata. Catalogue of American Amphibians and Reptiles.

[ref-17] Dinerstein E, Olson D, Joshi A, Vynne C, Burgess ND, Wikramanayake E, Hahn N, Palminteri S, Hedao P, Noss R, Hansen M, Locke H, Ellis EC, Jones B, Barber C, Hayes R, Kormos C, Martin V, Crist E, Sechrest W, Price L, Baillie JEM, Kindt R, Van Breugel P, Graudal L (2017). An ecoregion-based approach to protecting half the terrestrial realm. Bioscience.

[ref-18] Diniz-Filho JAF, Bini LM, Bastos RP, Vieira CM, Vieira LCG (2005). Priority areas for anuran conservation using biogeographical data: a comparison of greedy, rarity, and simulated annealing algorithms to define reserve networks in Cerrado. Brazilian Journal of Biology.

[ref-19] Dubeux MJM, Nascimento FA, Correia LL, Mott T (2022). DNA barcoding in Neotropical tadpoles: evaluation of 16S rRNA gene for the identification of anuran larvae from northeastern Brazil. Cuadernos de Herpetología.

[ref-20] Dubeux MJM, Nascimento FAC, Lima LR, Magalhães FM, Silva IRS, Gonçalves U, Almeida JPF, Correia LL, Garda AA, Mesquita DO, Rossa-Feres DC, Mott D (2020a). Morphological characterization and taxonomic key of tadpoles (Amphibia: Anura) from the northern region of the Atlantic Forest. Biota Neotropica.

[ref-21] Dubeux MJM, Silva TD, Mott T, Nascimento FAC (2020b). Redescription of the tadpole of *Leptodactylus natalensis* Lutz (Anura: Leptodactylidae), an inhabitat of the Brazilian Atlantic Forest. Zootaxa.

[ref-22] Dubois A (1995). Keratodont formulae in anuran tadpoles: proposal for standardisation. Journal of Zoological Systematics and Evolutionary Research.

[ref-23] Duellman WE, Marion AB, Hedges SB (2016). Phylogenetics, classification, and biogeography of the treefrogs (Amphibia: Anura: Arboranae). Zootaxa.

[ref-24] Felsenstein J (1985). Confidence limits on phylogenies: an approach using the bootstrap. Evolution.

[ref-25] Ferrão M, Moravec J, Ferreira AS, Moraes LJCL, Hanken J (2022). A new snouted treefrog of the genus *Scinax* (Anura, Hylidae) from the white-sand forests of central Amazonia. Breviora.

[ref-26] Fick SE, Hijmans RJ (2017). WorldClim 2: new 1km spatial resolution climate surfaces for global land areas. International Journal of Climatology.

[ref-27] França HS, Sá DP, Neto PGG, Lopes GN, Santos RJ, Andrade GV (2021). Anfíbios e répteis de Camaputiua e seus entornos.

[ref-28] Freitas MA, Vieira RS, Entiauspe-Neto OM, Sousa SO, Farias T, Sousa AG, Moura GJB (2017). Herpetofauna of the Northwest Amazon forest in the state of Maranhão, Brazil, with remarks on the Gurupi biological reserve. ZooKeys.

[ref-29] Frost DR (2023). Amphibian species of the world: an online reference. version 6.2 (20.09.2023). American Museum of Natural History, New York, USA. https://amphibiansoftheworld.amnh.org/index.php.

[ref-30] Gehara M, Crawford AJ, Orrico VGD, Rodríguez A, Lötters S, Fouquet A, Barrientos LS, Brusquetti F, De la Riva I, Ernesto R, Urrutia GG, Glaw F, Guayasamin JM, Holting M, Jansen M, Kok PJR, Kwet A, Lingnau R, Lira M, Moravec J, Pombal JP, Rojas-Runjaic FJM, Schulze A, Señaris JC, Solé M, Rodrigues MT, Twomey E, Haddad CFB, Vences M, Köhler J (2014). High levels of diversity uncovered in a widespread nominal taxon: continental phylogeography of the neotropical tree frog *Dendropsophus minutus*. PLOS ONE.

[ref-31] Gosner KL (1960). A simplified table for staging anuran embryo and larvae with notes on identification. Herpetologica.

[ref-32] Grosso JR, Pereyra MO, Candioti FV, Maciel NM, Baldo D (2020). Tadpoles of three species of the *Rhinella granulosa* group with a reinterpretation of larval characters. South American Journal of Herpetology.

[ref-33] Haas A, Das I (2011). Describing East Malaysian tadpole diversity: status and recommendations for standards and procedures associated with larval amphibian description and documentation. Bonner Zoologische Monographien.

[ref-34] Haddad CFB, Andrade GV, Cardoso AJ (1988). Anfíbios anuros do parque nacional da Serra da Canastra, estado de Minas Gerais. Brasil Florestal.

[ref-35] Haddad CFB, Toledo LF, Prado CPA, Loebmann D, Gasparini JL, Sazima I (2013). Guia de Anfíbios da Mata Atlântica: Diversidade e Biologia.

[ref-36] Hall TA (1999). BioEdit: a user-friendly biological sequence alignment editor and analysis program for Windows 95/98/NT. Nucleic Acids Symposium Series.

[ref-37] Hero J-M, Gascon C, Magnusson WE (1998). Direct and indirect effects of predation on tadpole community structure in the Amazon rainforest. Australian Journal of Ecology.

[ref-39] IBGE (2019). Instituto Brasileiro de Geografia e Estatística. Mapa de biomas do Brasil. https://agenciadenoticias.ibge.gov.br/agencia-sala-de-imprensa/2013-agencia-de-noticias/releases/25798-ibge-lanca-mapa-inedito-de-biomas-e-sistema-costeiro-marinho.

[ref-40] IMESC (2023). Relatório de Diversidade Faunística do Maranhão: avaliação da composição, áreas prioritárias, ameaças e recomendações de ações para sua conservação do Zoneamento Ecológico-Econômico do Estado do Maranhão (ZEE)–Etapa Bioma Cerrado e Sistema Costeiro.

[ref-41] IUCN (2021). The IUCN red list of threatened species. Version 2020-1. https://www.iucnredlist.org.

[ref-42] Jansen M, Bloch R, Schulze A, Pfenninger M (2011). Integrative inventory of Bolivia’s lowland anurans reveals hidden diversity. Zoologica Scripta.

[ref-43] Jetz W, Pyron RA (2018). The interplay of past diversification and evolutionary isolation with present imperilment across the amphibian tree of life. Nature Ecology and Evolution.

[ref-44] Jungfer KH, Faivovich J, Padial JM, Castroviejo-692 Fisher S, Lyra MM, Berneck B, Iglesias PP, Kok PJR, MacCulloch RD, Rodrigues MT, Verdade VK, Gastello CPT, Chaparro JC, Valdujo PH, Reichle S, Moravec J, Gvoždík V, Gagliardi-Urrutia G, Ernst R, De la Riva I, Means DB, Lima AP, Señaris JC, Wheeler WC, Haddad CFB (2013). Systematics of spiny-backed treefrogs (Hylidae: Osteocephalus): an Amazonian puzzle. Zoologica Scripta.

[ref-47] Köhler J, Venegas PJ, Castillo-Urbina E, Glaw F, Aguilar-Puntriano C, Vences M (2023). A third species of glassfrog in the genus *Chimerella* (Anura, Centrolenidae) from central Peru, discovered by an integrative taxonomic approach. Evolutionary Systematics.

[ref-45] Kopp K, Wachlevski M, Eterovick PC (2006). Environmental complexity reduces tadpole predation by water bugs. Canadian Journal of Zoology.

[ref-46] Kumar S, Stecher G, Li M, Knyaz C, Tamura K (2018). MEGA X: molecular evolutionary genetics analysis across computing platforms. Molecular Biology and Evolution.

[ref-48] Leite JMA, Sampaio JMS, Silva-Leite RR, Leite JRSA (2008). *Leptodactylus natalensis* (Lutz, 1930) (Amphibia, Anura, Leptodactylidae): first record from Maranhão state and new geographic distribution map. Biotemas.

[ref-49] Librado P, Rozas J (2009). DnaSPv5: a software for comprehensive analysis of DNA polymorphism data. Bioinformatics.

[ref-50] Lima LR, Dubeux MJM, do Nascimento FAC, Bruschi DP, Mott T (2019). Uncovering Neotropical treefrog diversity: integrative taxonomy reveals paraphyly in *Boana atlantica* (Amphibia, Anura, Hylidae). Amphibia-Reptilia.

[ref-51] Lopes GN, Serra RTA, Piorski NM, Andrade GV (2020). Pond characteristics influence the intraspecific variation in the morphometry of the tadpoles of two species of *Dendropsophus* (Anura: Hylidae) from the Cerrado savanna of northeastern Brazil. Anais da Academia Brasielira de Ciências.

[ref-62] Mângia S, de Magalhães FM, Leite FSF, Cavalheri DG, Garda AA (2022). A new species of *Proceratophrys* (Anura: Odontophrynidae) from Boqueirão da Onça, Northern Bahia State, Brazil. Journal of Herpetology.

[ref-52] Magalhães FM, Lyra ML, de Carvalho TR, Baldo D, Brusquetti F, Burella P, Colli GR, Gehara MC, Giaretta AA, Haddad CFB, Langone JA, López JA, Napoli MF, Santana DJ, de Sá RO, Garda AA (2020). Taxonomic review of South American butter frogs: phylogeny, geographic patterns, and species delimitation in the *Leptodactylus latrans* species group (Anura: Leptodactylidae). Herpetological Monographs.

[ref-53] Markmann M, Tautz D (2005). Reverse taxonomy: an approach towards determining the diversity of meiobenthic organisms based on ribosomal RNA signature sequences. Philosophical Transactions of the Royal Society B Biological Sciences.

[ref-54] Marques EQ, Marimon-Junior BH, Marimon BS, Matricardi EAT, Mews HA, Colli GR (2020). Redefining the Cerrado-Amazonia transition: implications for conservation. Biodiversity Conservation.

[ref-55] Martins MB, Oliveira TG (2011). Amazônia Maranhense: diversidade e conservação.

[ref-56] Matos RHR, Andrade GV, Hass A (2000). Reproductive biology and territoriality of *Phyllomedusa hypochondrialis* in Northeastern Brazil. Herpetological Review.

[ref-57] May PH, Anderson AB, Frazão JMF, Balick MJ (1985). Babassu palm in the agroforestry systems in Brazil’s Mid-North region. Agroforestry.

[ref-58] McDiarmid RW, Altig R (1999). Tadpoles. The biology of anuran larvae.

[ref-59] Mercês EA, Acuña Juncá F, Cousiño Casal FS (2009). Girinos de três espécies do gênero *Rhinella* Fitzinger, 1926 (Anura-Bufonidae), ocorrentes no estado da Bahia Brasil. Sitientibus Série Ciências Biológicas.

[ref-60] Mittermeier RA, Gil PR, Hoffmann M, Pilgrim J, Brooks, Mittermeier CG, Lamoreux J, Fonseca GAB (2005). Hotspots revisited: earth’s biologically richest and most endangered terrestrial ecoregions.

[ref-61] Myers N, Mittermeier RA, Mittermeier CG, Fonseca GAB, Kent J (2000). Biodiversity hotspots for conservation priorities. Nature.

[ref-63] Nori J, Lemes P, Urbina-Cardona N, Baldo D, Lescano J, Loyola R (2015). Amphibian conservation, land-use changes and protected areas: a global overview. Biological Conservation.

[ref-64] Orrico VGD, Grant T, Faivovich J, Rivera-Correa M, Rada M, Lyra ML, Cassini CS, Valdujo PH, Schargel WE, Machado DJ, Wheeler WC, Barrio-Amorós CL, Loebmann D, Moravec J, Zina J, Solé M, Sturaro MJ, Peloso PLV, Suárez P, Haddad CFB (2021). The phylogeny of Dendropsophini (Anura: Hylidae: Hylinae). Cladistics.

[ref-65] Pabijan M, Palomar G, Antunes B, Antoł W, Zieliński P, Babik W (2020). Evolutionary principles guiding amphibian conservation. Evolutionary Applications.

[ref-66] Palumbi S, Martin A, Romano S, McMillan WO, Stice L, Grabowski G (1991). The simple fool’s guide to PCR, version 2.0.

[ref-67] Pavan D, Moraes L (2023). Herpetofauna. Relatório de Diversidade Faunística do Maranhão: Avaliação da Composição, Áreas Prioritárias, Ameaças e Recomendações de Ações Para Sua Conservação do Zoneamento Ecológico-Econômico do Estado do Maranhão (ZEE)—Etapa Bioma Cerrado e Sistema Costeiro.

[ref-68] Pezzuti TL, Leite FSF, Garcia PCA (2019). Chave de identificação interativa para os girinos do Quadrilátero Ferrífero, Minas Gerais, Sudeste do Brasil. Versão 1.0. Viçosa: Universidade Federal de Viçosa. http://biodiversus.com.br/saglab/aqf/chave/girinos/.

[ref-69] Pezzuti TL, Leite FSF, Rossa-Feres DC, Garcia PCA (2021). The tadpoles of the iron quadrangle, Southerastern Brazil: a baseline for larval knowledge and Anuran conservation in a diverse and threatened region. South American Journal of Herpetology.

[ref-70] Provete DB, Garey MV, Silva FR, Jordani MX (2012). Knowledge gaps and bibliographical revision about descriptions of free-swimming anuran larvae from Brazil. North-Western Journal of Zoology.

[ref-72] Ramos EKS, Magalhães RF, Marques NCS, Baêta D, Garcia PCA, Santos FR (2019). Cryptic diversity in Brazilian endemic monkey frogs (Hylidae, Phyllomedusinae, *Pithecopus*) revealed by multispecies coalescent and integrative approaches. Molecular Phylogenetics and Evolution.

[ref-73] Randrianiaina RD, Strauß A, Glos J, Glaw F, Vences M (2011). Diversity, external morphology and reverse taxonomy in the specialized tadpoles of Malagasy river bank frogs of the subgenus *Ochthomantis* (genus *Mantidactylus*). Contributions to Zoology.

[ref-74] Randrianiaina RD, Strauß A, Glos J, Vences M (2012). Diversity of the strongly rheophilous tadpoles of Malagasy tree frogs, genus *Boophis* (Anura, Mantellidae), and identification of new candidate species via larval DNA sequence and morphology. ZooKeys.

[ref-75] Relyea RA, Werner EE (2000). Morphological plasticity in four larval anurans distributed along an environmental gradient. Copeia.

[ref-76] Ricklefs RE, Relyea R (2013). Ecology. The economy of nature.

[ref-77] Rossa-Feres DC, Nomura F (2006). Characterization and taxonomic key for tadpoles (Amphibia: Anura) from the northwestern region of São Paulo State, Brazil. Biota Neotropica.

[ref-78] Ruggeri J, Toledo LF, Carvalho-e-Silva SP (2018). Stream tadpoles present high prevalence but low infection loads of *Batrachochytrium dendrobatidis* (Chytridiomycota). Hydrobiologia.

[ref-79] Santos MCO, Lima MSCS, Souza PS, Silva IC, Pederassi J (2014). Geographic distribution: *Leptodactylus chaquensis*. Herpetological Review.

[ref-80] Schulze A, Jansen M, Köhler G (2015). Tadpole diversity of Bolivia’s lowland anuran communities: molecular identification, morphological characterization, and ecological assignment. Zootaxa.

[ref-81] Segalla MV, Berneck B, Canedo C, Caramaschi U, Cruz CAG, Garcia PCA, Grant T, Haddad CFB, Lourenço ACC, Mângia S, Mott T, Nascimento LB, Toledo LF, Langone JA (2021). List of Brazilian Amphibians. Herpetologia Brasileira.

[ref-82] Silva HSVP, Santos CL, Pereira SRF, Luvizotto-Santos R, Andrade GV, Nunes GS (2013). Toxicidade aguda e genotoxicidade do agrotóxico comercial Folisuper 600BR a girinos de *Physalaemus cuvieri* (Anura: Leiuperidae). Pesticidas.

[ref-83] Silva-Alves VD, D’Ávila RS, Costa TM, Barbosa APD, Brum BR, Silva CPA, Ignácio Á.RA, Carniello MA, Muniz CC, Santos-Filho M, Seba MFR, Nogueira OMA, Silva JSH, Gusmão AC, Mudrek JR, Silva OD, Silva DJ, Canale GR (2019). Geographic range extension of *Elachistocleis corumbaensis* Piva, Caramaschi & Albuquerque, 2017 (Anura, Microhylidae) with new records in ecotonal zones in the state of Mato Grosso Brazil. Check List.

[ref-84] Skelly DK (1997). Tadpole communities: pond permanence and predation are powerful forces shaping the structure of tadpole communities. American Scientist.

[ref-85] Streicher JW, Sadler R, Loader SP (2020). Amphibian taxonomy: early 21^st^ century case studies. Journal of Natural History.

[ref-86] Tamura K, Nei M (1993). Estimation of the number of nucleotide substitutions in the control region of mitochondrial DNA in humans and chimpanzees. Molecular Biology and Evolution.

[ref-87] Thompson JD, Higgins DG, Gibson TJ (1994). CLUSTAL W: improving the sensitivity of progressive multiple sequence alignment through sequence weighting, position-specific gap penalties and weight matrix choice. Nucleic Acids Research.

[ref-88] Vera Candioti MF (2007). Anatomy of anuran tadpoles from lentic water bodies: systematic relevance and correlation with feeding habits. Zootaxa.

[ref-89] Vieira Serra FC, Bezerra Almeida E (2021). Phytosociology, successional level, and conservation of the woody component in a “restinga” of Maranhão island, Brazi. Revista de Biología Tropical.

