# Supplementary material for: Discovering the diversity of tadpoles in the mid-north Brazil: morphological and molecular identification, and characterization of the habitat
Source: PeerJ. 2023 Dec 14;11:e16640. doi: 10.7717/peerj.16640 (PMC10725668; doi:10.7717/peerj.16640)
Supplement: Supplemental Information 1 — Annual Temp. = annual temperature average (o C); Anual Rain. = annual rainfall average (mm); Season of sampling = rainy season from December to April, dry season from May to November. Most of the sampling points presented in Appendix S1 were sampled only once, except points 6, 9 and 10 that were sampled twice each. [file peerj-11-16640-s001.docx]

## Discovering the diversity of tadpoles in the mid-north Brazil: morphological and molecular identification, and characterization of the habitat

Patrícia dos Santos Sousa^1^, Carlos Augusto Silva de Azevêdo^1^, Maria Claudene Barros^1^, Elmary da Costa Fraga^1^, Thaís B. Guedes^2,3^

^1^Centro de Estudos Superiores de Caxias, Universidade Estadual do Maranhão, 65604-380, Caxias, MA, Brazil

^2^Departamento de Biologia Animal, Instituto de Biologia, Universidade Estadual de Campinas, 13083-862, Campinas, SP, Brazil

^3^Gothenburg Global Biodiversity Center, University of Gothenburg, Department of Biological and Environmental Sciences, Box 461, SE-405-30, Göteborg, Sweden

Corresponding author: Thaís B. Guedes. Address: Rua Monteiro Lobato, 255, Cidade Universitária, 13083-862, Campinas, SP, Brazil. E-mail: thaisbguedes@yahoo.com.br

Supporting information

**Appendix S1.** Spatial and climatic data (extracted from WorldClim: Fick & Hijmans, 2017) of the study area in the eastern Maranhão, mid-north region of Brazil. Annual Temp. = annual temperature average (^o^ C); Anual Rain. = annual rainfall average (mm); Season of sampling = rainy season from December to April, dry season from May to November. Most of the sampling points presented in Appendix S1 were sampled only once, except points 6, 9 and 10 that were sampled twice each.

| **Sampled localities** | **Point** | **Geographic coordinates** | **Annual Temp. (min/max)** | **Annual Rain.** | **Season of sampling** |
| --- | --- | --- | --- | --- | --- |
|  |  |  |  |  | Rainy |
| Village of Timbaúba, municipality of São Mateus do Maranhão, Maranhão, Brazil | 1 | 4°06'33.1"S  44°28'20.0"W | 22.1/32.7 | 132.5 |  |
|  |  |  |  |  | Rainy |
| Village of São José, municipality of São Mateus do Maranhão, Maranhão, Brazil | 2 | 3°58'45.8"S  44°26'39.6"W | 22.1/32.6 | 138.9 |  |
|  |  |  |  |  | Rainy |
| Village of Nego, municipality of Coroatá, Maranhão, Brazil | 3 | 4°2'46.11"S  44°4'16.54"W | 21.9/32.4 | 133.5 |  |
|  |  |  |  |  | Dry |
| Village of Cheio d'água, municipality of Aldeias Altas, Maranhão, Brazil | 4 | 4°38'37.1"S 43°30'45.1"W | 21.5/32.7 | 128.3 |  |
|  |  |  |  |  | Dry |
| Surroundings of Buriti Corrente, municipality of Caxias, Maranhão, Brazil | 5 | 4°45'00.8"S 43°40'26.7"W | 21.5/32.7 | 126 |  |
|  |  |  |  |  | Rainy |
| Surroundings of Inhamum Protected Area, municipality of Caxias, Maranhão, Brazil | 6 | 4°53'23.0"S 43°24'30.9"W | 21.3/32.4 | 127.6 |  |
|  |  |  |  |  | Dry |
| Surroundings of Buriti do Meio Protected Area, municipality of Caxias, Maranhão, Brazil | 7 | 4°54'15.3"S 43°06'56.4"W | 21.5/32.8 | 130.4 |  |
| Village of Sobra, municipality of Caxias, Maranhão, Brazil | 8 | 5°02'44.2"S  43°34'14.7"W |  | 128.6 | Dry |
|  |  |  | 21.2/32.1 |  |  |
| Village of Bacabal, municipality of São João do Sóter, Maranhão, Brazil | 9 | 5°07'53.5"S  43°48'13.8"W | 21.1/32.3 | 116.5 | Rainy |

| Village of São José dos Perdidos, municipality de São João do Sóter,  Maranhão, Brazil | 10 | 5°10'39.4"S 43°51'59.2"W | 21.1/32.2 | 114.3 | Rainy |
| --- | --- | --- | --- | --- | --- |
