# Supplementary material for: Discovering the diversity of tadpoles in the mid-north Brazil: morphological and molecular identification, and characterization of the habitat
Source: PeerJ. 2023 Dec 14;11:e16640. doi: 10.7717/peerj.16640 (PMC10725668; doi:10.7717/peerj.16640)
Supplement: Supplemental Information 2 — (A) Map of Maranhão highlighting the location of the sampling areas in the following municipalities: São Mateus (1 and 2), Coroatá (3), Aldeias Altas (4), Caxias (5, 6, 7, 8), and São João do Sóter (9, 10). Municipality and biome limits follow (IBGE, 2019). [file peerj-11-16640-s002.docx]

## Discovering the diversity of tadpoles in the mid-north Brazil: morphological and molecular identification, and characterization of the habitat

Patrícia dos Santos Sousa^1^, Carlos Augusto Silva de Azevêdo^1^, Maria Claudene Barros^1^, Elmary da Costa Fraga^1^, Thaís B. Guedes^2,3^

^1^Centro de Estudos Superiores de Caxias, Universidade Estadual do Maranhão, 65604-380, Caxias, MA, Brazil

^2^Departamento de Biologia Animal, Instituto de Biologia, Universidade Estadual de Campinas, 13083-862, Campinas, SP, Brazil

^3^Gothenburg Global Biodiversity Center, University of Gothenburg, Department of Biological and Environmental Sciences, Box 461, SE-405-30, Göteborg, Sweden

Corresponding author: Thaís B. Guedes. Address: Rua Monteiro Lobato, 255, Cidade Universitária, 13083-862, Campinas, SP, Brazil. E-mail: thaisbguedes@yahoo.com.br

Supporting information


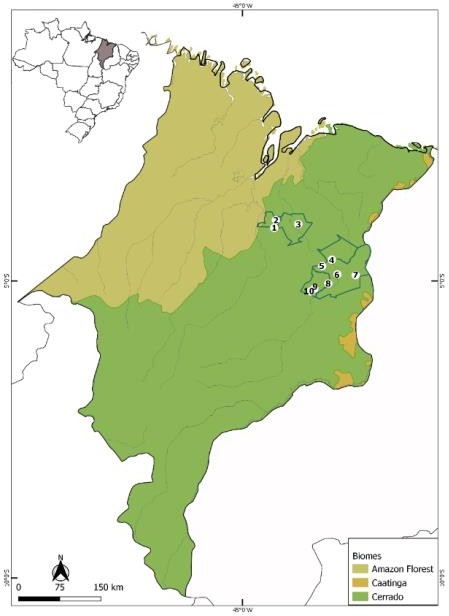


**Appendix S2.** View of the study area in the eastern Maranhão, mid-north region of Brazil. (A) Map of Maranhão highlighting the location of the sampling areas in the following municipalities: São Mateus do Maranhão (1 and 2), Coroatá (3), Aldeias Altas (4), Caxias (5, 6, 7, 8), and São João do Sóter (9, 10). Municipal and biome limits follow (IBGE, 2019).
