# Supplementary material for: Discovering the diversity of tadpoles in the mid-north Brazil: morphological and molecular identification, and characterization of the habitat
Source: PeerJ. 2023 Dec 14;11:e16640. doi: 10.7717/peerj.16640 (PMC10725668; doi:10.7717/peerj.16640)
Supplement: Supplemental Information 3 [file peerj-11-16640-s003.docx]

## Discovering the diversity of tadpoles in the mid-north Brazil: morphological and molecular identification, and characterization of the habitat

Patrícia dos Santos Sousa^1^, Carlos Augusto Silva de Azevêdo^1^, Maria Claudene Barros^1^, Elmary da Costa Fraga^1^, Thaís B. Guedes^2,3^

^1^Centro de Estudos Superiores de Caxias, Universidade Estadual do Maranhão, 65604-380, Caxias, MA, Brazil

^2^Departamento de Biologia Animal, Instituto de Biologia, Universidade Estadual de Campinas, 13083-862, Campinas, SP, Brazil

^3^Gothenburg Global Biodiversity Center, University of Gothenburg, Department of Biological and Environmental Sciences, Box 461, SE-405-30, Göteborg, Sweden

Corresponding author: Thaís B. Guedes. Address: Rua Monteiro Lobato, 255, Cidade Universitária, 13083-862, Campinas, SP, Brazil. E-mail: thaisbguedes@yahoo.com.br

Supporting information

**Appendix S3.** Voucher numbers for the specimens of the present study (organized by lots considering collection data, locality and species) housed at the Museum of Biological Diversity – Zoology area (MDBio – ZUEC), of the Institute of Biology, State University of Campinas, Brazil.

| **ZUEC** | **Species** | **Date** | **ZUEC** | **Species** | **Date** |
| --- | --- | --- | --- | --- | --- |
| 26120 | *Boana* cf. *atlantica* | 12.x.2019 | 26195 | *Leptodactylus natalensis* | 27.xii.2019 |
| 26121 | *Boana* cf. *atlantica* | 24.x.2019 | 26197 | *Leptodactylus fuscus* | 03.i.2020 |
| 26122 | *Rhinella diptycha* | 25.xii.2019 | 26198 | *Leptodactylus fuscus* | 29.i.2020 |
| 26123 | *Dermatonotus muelleri* | 12.i.2020 | 26199 | *Leptodactylus fuscus* | 30.i.2020 |
| 26124 | *Dermatonotus muelleri* | 09.i.2020 | 26200 | *Leptodactylus fuscus* | 02.ii.2020 |
| 26125 | *Dermatonotus muelleri* | 06.i.2020 | 26201 | *Scinax* cf. *similis* | 07.iv.2019 |
| 26126 | *Dermatonotus muelleri* | 07.i.2020 | 26202 | *Scinax* cf. *similis* | 19.iii.2019 |
| 26127 | *Rhinella diptycha* | 12.i.2020 | 26203 | *Physalaemus nattereri* | 27.xi.2019 |
| 26128 | *Rhinella diptycha* | 09.i.2020 | 26204 | *Physalaemus nattereri* | 02.ii.2020 |
| 26129 | *Leptodactylus vastus* | 01.i.2020 | 26205 | *Osteocephalus taurinus* | 25.xii.2019 |
| 26130 | *Scinax x-signatus* | 20.ix.2019 | 26206 | *Osteocephalus taurinus* | 24.x.2019 |
| 26131 | *Scinax x-signatus* | 28.i.2020 | 26207 | *Boana multifasciata* | 28.viii.2019 |
| 26132 | *Scinax x-signatus* | 07.vi.2019 | 26208 | *Boana multifasciata* | 12.x.2019 |
| 26133 | *Rhinella diptycha* | 13.x.2019 | 26209 | *Boana multifasciata* | 25.ii.2019 |
| 26134 | *Physalaemus cuvieri* | 30.i.2020 | 26210 | *Boana multifasciata* | 24.x.2019 |
| 26135 | *Elachistocleis cesarii* | 30.i.2020 | 26211 | *Scinax x-signatus* | 04.x.2019 |
| 26136 | *Leptodactylus vastus* | 30.i.2019 | 26212 | *Scinax x-signatus* | 30.i.2020 |
| 26137 | *Leptodactylus pustulatus* | 20.ix.2019 | 26213 | *Scinax x-signatus* | 02.ii.2020 |
| 26138 | *Pseudopaludicola* sp. | 20.ix.2019 | 26214 | *Scinax x-signatus* | 21.xii.2019 |
| 26139 | *Scinax fuscomarginatus* | 28.i.2020 | 26215 | *Trachycephalus typhonius* | 09.i.2020 |
| 26140 | *Physalaemus cuvieri* | 02.ii.2020 | 26216 | *Trachycephalus typhonius* | 12.i.2020 |
| 26141 | *Physalaemus cuvieri* | 19.iii.2019 | 26217 | *Trachycephalus typhonius* | 04.i.2020 |
| 26142 | *Physalaemus cuvieri* | 07.iv.2019 | 26218 | *Dendropsophus soaresi* | 30.i.2020 |
| 26143 | *Physalaemus cuvieri* | 07.vi.2019 | 26219 | *Dendropsophus soaresi* | 19.iii.2019 |
| 26144 | *Rhinella diptycha* | 16.xi.2019 | 26220 | *Dendropsophus soaresi* | 12.xii.2019 |
| 26145 | *Leptodactylus vastus* | 19.iii.2019 | 26221 | *Dendropsophus soaresi* | 12.i.2020 |
| 26146 | *Leptodactylus vastus* | 03.i.2020 | 26222 | *Dendropsophus soaresi* | 01.i.2020 |
| 26147 | *Physalaemus cuvieri* | 07.vi.2019 | 26223 | *Dendropsophus soaresi* | 02.ii.2020 |
| 26148 | *Boana raniceps* | 07.vi.2019 | 26224 | *Dendropsophus soaresi* | 09.i.2020 |
| 26149 | *Dendropsophus nanus* | 07.vi.2019 | 26225 | *Dendropsophus soaresi* | 15.i.2020 |
| 26150 | *Boana* cf. *atlantica* | 20.ix.2019 | 26226 | *Leptodactylus mystaceus* | 01.xii.2019 |
| 26189 | *Pithecopus* aff. *hypochondrialis* | 19.iii.2019 | 26227 | *Leptodactylus macrosternum* | 04.i.2020 |
| 26190 | *Pithecopus* aff. *hypochondrialis* | 02.ii.2020 | 26228 | *Leptodactylus macrosternum* | 06.i.2020 |
| 26191 | *Pithecopus* aff. *hypochondrialis* | 07.vi.2019 | 26229 | *Leptodactylus macrosternum* | 09.i.2020 |
| 26192 | *Leptodactylus natalensis* | 10.vi.2019 | 26230 | *Rhinella mirandaribeiroi* | 21.xii.2019 |
| 26193 | *Pithecopus* aff. *hypochondrialis* | 07.iv.2019 | 26231 | *Rhinella mirandaribeiroi* | 07.i.2020 |
| 26194 | *Pithecopus* aff. *hypochondrialis* | 19.iii.2019 | 26232 | *Rhinella mirandaribeiroi* | 09.i.2020 |
