# Supplementary material for: Discovering the diversity of tadpoles in the mid-north Brazil: morphological and molecular identification, and characterization of the habitat
Source: PeerJ. 2023 Dec 14;11:e16640. doi: 10.7717/peerj.16640 (PMC10725668; doi:10.7717/peerj.16640)
Supplement: Supplemental Information 4 [file peerj-11-16640-s004.docx]

## Discovering the diversity of tadpoles in the mid-north Brazil: morphological and molecular identification, and characterization of the habitat

Patrícia dos Santos Sousa^1^, Carlos Augusto Silva de Azevêdo^1^, Maria Claudene Barros^1^, Elmary da Costa Fraga^1^, Thaís B. Guedes^2,3^

^1^Centro de Estudos Superiores de Caxias, Universidade Estadual do Maranhão, 65604-380, Caxias, MA, Brazil

^2^Departamento de Biologia Animal, Instituto de Biologia, Universidade Estadual de Campinas, 13083-862, Campinas, SP, Brazil

^3^Gothenburg Global Biodiversity Center, University of Gothenburg, Department of Biological and Environmental Sciences, Box 461, SE-405-30, Göteborg, Sweden

Corresponding author: Thaís B. Guedes. Address: Rua Monteiro Lobato, 255, Cidade Universitária, 13083-862, Campinas, SP, Brazil. E-mail: thaisbguedes@yahoo.com.br

Supporting information

**Appendix S4.** GenBank access numbers and sequences.

MZ572904 Seq4 Rhinella diptycha

MZ572901 Seq1 Rhinella mirandaribeiroi

MZ572902 Seq2 Rhinella mirandaribeiroi

MZ572903 Seq3 Rhinella mirandaribeiroi

MZ570071 Seq1 Boana cf. atlantica

MZ568626 Seq1 Boana multifasciata

MZ568627 Seq2 Boana multifasciata

MZ568628 Seq3 Boana multifasciata

MZ568629 Seq4 Boana multifasciata

MZ572866 Seq1 Dendropsophus soaresi

MZ572867 Seq2 Dendropsophus soaresi

MZ572868 Seq3 Dendropsophus soaresi

MZ572869 Seq4 Dendropsophus soaresi

MZ572887 Seq1 Osteocephalus taurinus

MZ572888 Seq2 Osteocephalus taurinus

MZ578452 Seq1 Pithecopus aff. hypochondrialis

MZ578453 Seq2 Pithecopus aff. hypochondrialis

MZ578454 Seq3 Pithecopus aff. hypochondrialis

MZ578455 Seq4 Pithecopus aff. hypochondrialis

MZ578456 Seq5 Pithecopus aff. hypochondrialis

MZ572905 Seq1 Scinax fuscomarginatus

MZ572906 Seq2 Scinax fuscomarginatus

MZ572907 Seq3 Scinax fuscomarginatus

MZ578445 Seq1_1 Scinax cf. nebulosus

MZ578446 Seq2_1 Scinax cf. nebulosus

MZ578442 Seq1_3 Scinax cf. similis

MZ578443 Seq2_3 Scinax cf. similis

MZ578447 Seq1_2 Scinax x-signatus

MZ578448 Seq2_2 Scinax x-signatus

MZ578449 Seq3_1 Scinax x-signatus

MZ578450 Seq4_1 Scinax x-signatus

MZ578451 Seq5_1 Scinax x-signatus

MZ572889 Seq3 Trachycephalus typhonius

MZ572883 Seq2 Leptodactylus fuscus

MZ572882 Seq1 Leptodactylus macrosternum

MZ572875 Seq1 Leptodactylus mystaceus

MZ572876 Seq2 Leptodactylus mystaceus

MZ572877 Seq3 Leptodactylus mystaceus

MZ572878 Seq1 Leptodactylus natalensis

MZ572879 Seq2 Leptodactylus natalensis

MZ572880 Seq3 Leptodactylus natalensis

MZ572881 Seq4 Leptodactylus natalensis

MZ572884 Seq3 Leptodactylus pustulatus

MZ572885 Seq4 Leptodactylus troglodytes

MZ572890 Seq1 Physalaemus cuvieri

MZ572891 Seq2 Physalaemus cuvieri

MZ572892 Seq3 Physalaemus cuvieri

MZ572893 Seq4 Physalaemus cuvieri

MZ572894 Seq5 Physalaemus cuvieri

MZ572895 Seq6 Physalaemus cuvieri

MZ572896 Seq1 Physalaemus nattereri

MZ572897 Seq2 Physalaemus nattereri

MZ572898 Seq3 Physalaemus nattereri

MZ572899 Seq4 Physalaemus nattereri

MZ572900 Seq5 Physalaemus nattereri

MZ572873 Seq2 Dermatonotus muelleri

MZ572872 Seq1 Elachistocleis cesarii

>Seq1 [organism-Boana multifasciata] Boana multifasciata 16S ribosomal RNA, partial cds; mitochondrial.

cCGCGGTATCCTAACCGTGCGAAGGTAGCGTAATCACTTGTTCTTTAAATGAGGACTAGTATGAATGGCACCACGAGGGTTAAGCTGTCTCCTTTTTCTAATCAGTGAAACTAATCTCCCCGTGAAGAAGCGGGGATCACCCTATAAGACGAGAAGACCCTATGGAGCTTAAAACAAATTAACACCTGCTTCTATCCACTCTCCTTCCTGAATTAACACATAAAATATAGCACCCTTGATTAATAGTTTTCGATTGGGGTGATCATGGAGCAAAAACCAACCTCCACGACGAAAAGGAGCACTTCCCTGAGCTAAAAGCTACAACTTTAAGCACTAAAAAATTAACGCACTTTGACCCAAATTTTTGAGCAACGAACCAAGTTACCCTAGGGATAACAGCGCAATCCATTTCAAGAGCCCCTATCGACAAATGGGTTTACGACCTCGATGTTGGATCAGGGTATCCTAGTGGTGCAGCCGCTACTAACGGTTCGTTTGTTCAACGATTAAAACCCTACGTGATCTGAGTTCAGACCGGA

>Seq2 [organism-Boana multifasciata] Boana multifasciata 16S ribosomal RNA, partial cds; mitochondrial.

CCGCGGTATCCTAACCGTGCGAAGGTAGCGTAATCACTTGTTCTTTAAATGAGGACTAGTATGAATGGCACCACGAGGGTTAAGCTGTCTCCTTTTTCTAATCAGTGAAACTAATCTCCCCGTGAAGAAGCGGGGATCACCCTATAAGACGAGAAGACCCTATGGAGCTTAAAACAAATTAACACCTGCTTCTATCCACTCTCCTTCCTGAATTAACACATAAAATATAGCACCCTTGATTAATAGTTTTCGATTGGGGTGATCATGGAGCAAAAACCAACCTCCACGACGAAAAGGAGCACTTCCCTGAGCTAAAAGCTACAACTTTAAGCACTAAAAAATTAACGCACTTTGACCCAAATTTTTGAGCAACGAACCAAGTTACCCTAGGGATAACAGCGCAATCCATTTCAAGAGCCCCTATCGACAAATGGGTTTACGACCTCGATGTTGGATCAGGGTATCCTAGTGGTGCAGCCGCTACTAACGGTTCGTTTGTTCAACGATTAAAACCCTACGTGATCTGAGTTCAGACCGGA

>Seq3 [organism-Boana multifasciata] Boana multifasciata 16S ribosomal RNA, partial cds; mitochondrial.

CCGCGGTATCCTAACCGTGCGAAGGtAGCGtAATCACTTGTTCTTTAAATGAGGACTAGTATGAATGGCACCACGAGGGTTAAGCTGTCTCCTTTTTCTAATCAGTGAAACTAATCTCCCCGTGAAGAAGCGGGGATCACCCTATAAGACGAGAAGACCCTATGGAGCTTAAAACAAATTAACACCTGCTTCTATCCACTCTCCTTCCTGAGTTAACACATAAAATATAGCACCCTTGATTAATAGTTTTCGATTGGGGTGATCATGGAGCAAAAACCAACCTCCACGACGAAAAGGAGCACTTCCCTGAGCTAAAAGCTACAACTTTAAGCACTAAAAAATTAACGCACTTTGACCCAAATTTTTGAGCAACGAACCAAGTTACCCTAGGGATAACAGCGCAATCCATTTCAAGAGCCCCTATCGACAAGTGGGTTTACGACCTCGATGTTGGATCAGGGTATCCTAGTGGTGCAGCCGCTACTAACGGTTCGTTTGTTCAACGATTAAAACCCTACGTGATCTGAGTTCAGACCGGA

>Seq4 [organism-Boana multifasciata] Boana multifasciata 16S ribosomal RNA, partial cds; mitochondrial.

CCGCGGTATCCTAACCGTGCGAAGGTAGCGTAATCACTTGTTCTTTAAATGAGGACTAGTATGAATGGCACCACGAGGGTTAAGCTGTCTCCTTTTTCTAATCAGTGAAACTAATCTCCCCGTGAAGAAGCGGGGATCACCCTATAAGACGAGAAGACCCTATGGAGCTTAAAACAAATTAACACCTGCTTCTATCCACTCTCCTTCCTGAATTAACACATAAAATATAGCACCCTTGATTAATAGTTTTCGATTGGGGTGATCATGGAGCAAAAACCAACCTCCACGACGAAAAGGAGCACTTCCCTGAGCTAAAAGCTACAACTTTAAGCACTAAAAAATTAACGCACTTTGACCCAAATTTTTGAGCAACGAACCAAGTTACCCTAGGGATAACAGCGCAATCCATTTCAAGAGCCCCTATCGACAAATGGGTTTACGACCTCGATGTTGGATCAGGGTATCCTAGTGGTGCAGCCGCTACTAACGGTTCGTTTGTTCAACGATTAAAACCCTACGTGATCTGAGTTCAGACCGGA

>Seq1 [organism-Boana cf. atlantica] Boana cf. atlantica 16S ribosomal RNA, partial cds; mitochondrial.

CCGCGGTATCCTAACCGTGCGAAGGTAGCGTAATCACTTGTTCTTTAAATGAGGACTAGTATGAACGGCACCACGAAGGTTATACTGTCTCCTTTCTCTAATCAGTAAAACTAATCTCCCCGTGAAGAAGCGAGGATAAATTTATAAGACGAGAAGACCCTATGGAGCTTTAAACTAACCAGCATTTGCTATATACAAAATTTTTAACAACTTTAAAGATAATCTAGCCCTCTGACTGGTAATTTTCGATTGGGGTGATCACGGAATAAAAATAACCTCCGTGTCGAAAGGGCTTACCTGAGCAAAAAGCGACAGCTTTCCGCACTAAAAAATTAACGTTTATTGACCCAATATTTTGATCAACGAACCAAGTTACCCTAGGGATAACAGCGCAATCTACTTCAAGAGTCCCTATCGACAAGTAGGTTTACGACCTCGATGTTGGATCAGGGTACCCCAGTGGTGCAGCCGCTACTAAAGGTTCGTTTGTTCAACGATTAATACCCTACGTGATCTGAGTTCAGACCGGA

>Seq1 [organism-Dendropsophus soaresi] Dendropsophus soaresi 16S ribosomal RNA, partial cds; mitochondrial.

CCGCGGTATCCTAACCGTGCGAAGGTAGCGTAATCACTTGTTCTTTAAATGAGGACTAGTATGAATGGCACCACGAGGGTTGTACTGTCTCCTTTTTCTAATCAATGAAACTGATCTCCCCGTGAAGAAGCGGGAATATAAATATAAGACGAGAAGACCCTATGGAGCTTTAAATTTTATTACACTGACTTATTATAAAGCAAAGTTTCAGAACAATAAAAGCTATTTTAGTCGGAATGATTATAAATTTTAGGTTGGGGTGACCGCGGAGAAAAAATTATCCTCCACATTGAACGGAGCTACTCCTGAGCGAAAAACTACAACTTTAAGCACCAATAAATTGACATTAATTGACCCAATATATTGATCAACGAACCAAGTTACCCTAGGGATAACAGCGCAATCCACCTCAAGAGCCCTTATCGACAGGTGGGTTTACGACCTCGATGTTGGATCAGGGCATCCTAGTGGTGCAGCCGCTACTAAAGGTTCGTTTGTTCAACGATTAAAGCCCTACGTGATCTGAGTTCAGACCGGA

>Seq2 [organism-Dendropsophus soaresi] Dendropsophus soaresi 16S ribosomal RNA, partial cds; mitochondrial.

CCGCGGTATCCTAACCGTGCGAAGGTAGCGTAATCACTTGTTCTTTAAATGAGGACTAGTATGAATGGCACCACGAGGGTTGTACTGTCTCCTTTTTCTAATCAATGAAACTGATCTCCCCGTGAAGAAGCGGGGATATAAATATAAGACGAGAAGACCCTATGGAGCTTTAAATTTTATTACACTGACTTATTATAAAGCAAAGTTTCAGAACAATAAAAGCTATTTTAGTCGGAATGATTATAAATTTTAGGTTGGGGTGACCGCGGAGAAAAAATTATCCTCCATATTGAACGGAGCTACTCCTGAGCGAAAAACTACAACTTTAAGCACCAATAAATTGACATTAATTGACCCAATATATTGATCAACGAACCAAGTTACCCTAGGGATAACAGCGCAATCCACCTCAAGAGCCCTTATCGACAGGTGGGTTTACGACCTCGATGTTGGATCAGGGCATCCTAGTGGTGCAGCCGCTACTAAAGGTTCGTTTGTTCAACGATTAAAGCCCTACGTGATCTGAGTTCAGACCGGA

>Seq3 [organism-Dendropsophus soaresi] Dendropsophus soaresi 16S ribosomal RNA, partial cds; mitochondrial.

CCGCGGTATCCTAACCGTGCGAAGGTAGCGTAATCACTTGTTCTTTAAATGAGGACTAGTATGAATGGCACCACGAGGGTTGTACTGTCTCCTTTTTCTAATCAATGAAACTGATCTCCCCGTGAAGAAGCGGGGATATAAATATAAGACGAGAAGACCCTATGGAGCTTTAAATTTTATTACACTGACTTATTATAAAGCAAAGTTTCAGAACAATAAAAGCTATTTTAGTCGGAATGATTATAAATTTTAGGTTGGGGTGACCGCGGAGAAAAAATTATCCTCCACATTGAACGGAGCTACTCCTGAGCGAAAAACTACAACTTTAAGCACCAATAAATTGACATTAATTGACCCAATATATTGATCAACGAACCAAGTTACCCTAGGGATAACAGCGCAATCCACCTCAAGAGCCCTTATCGACAGGTGGGTTTACGACCTCGATGTTGGATCAGGGCATCCTAGTGGTGCAGCCGCTACTAAAGGTTCGTTTGTTCAACGATTAAAGCCCTACGTGATCTGAGTTCAGACCGGA

>Seq4 [organism-Dendropsophus soaresi] Dendropsophus soaresi 16S ribosomal RNA, partial cds; mitochondrial.

CCGCGGTATCCTAACCGTGCGAAGGTAGCGTAATCACTTGTTCTTTAAATGAGGACTAGTATGAATGGCACCACGAGGGTTGTACTGTCTCCTTTTTCTAATCAATGAAACTGATCTCCCCGTGAAGAAGCGGGGATATAAATATAAGACGAGAAGACCCTATGGAGCTTTAAATTTTATTACACTGACTTATTATAAAGCAAAGTTTCAGAACAATAAAAGCTATTTTAGTCGGAATGATTATAAATTTTAGGTTGGGGTGACCGCGGAGAAAAAATTATCCTCCACATTGAACGGAGCTACTCCTGAGCGAAAAACTACAACTTTAAGCACCAATAAATTGACATTAATTGACCCAATATATTGATCAACGAACCAAGTTACCCTAGGGATAACAGCGCAATCCACCTCAAGAGCCCTTATCGACAGGTGGGTTTACGACCTCGATGTTGGATCAGGGCATCCTAGTGGTGCAGCCGCTACTAAAGGTTCGTTTGTTCAACGATTAAAGCCCTACGTGATCTGAGTTCAGACCGGA

>Seq1 [organism-Elachiastocheis cesarrie] Elachiastocheis cesarrie 16S ribosomal RNA, partial cds; mitochondrial.

CCGCGGTACCCTAACCGTGCGAAGGTAGCGTAATCACTTGTTCTTTAAATGAGGACTAGTATGAACGGCATCACGAGGGTTGCACTGTCTCCCACTTCTAATCAGTGAAACTGATCCCCCCGTGAAGAAGCGGGGATAAACCTATAAGACGAGAAGACCCCATGGAGCTTTAAACTCAGTACCAATCACCTTAAATCACACCACCATAACCCGGTGTCTCTGGCTACTAGTTTTAGGTTGGGGTGACCACGGAGCATAATAAAACCTCCATGTTGAAAACTTCCTCTAATCCAAGAGTGACAACTCTAAGAATCAAAAACTGACATACATTGACCCAAATATTTTGATCAACGAACCAAGTTACCCTGGGGATAACAGCGCAATCCATTTCAAGAGCTCCTATCGACAAATGGGTTTACGACCTCGATGTTGGATCAGGGTATCCCAGTGGTGCAGCCGCTACTAAAGGTTCGTTTGTTCAACGATTAAAACCCTACGTGATCTGAGTTCAGACCGGA

>Seq2 [organism-Dermatonotus muelleri] Dermatonotus muelleri 16S ribosomal RNA, partial cds; mitochondrial.

CCGCGGTACCCTAACCGTGCGAAGGTAGCGTAATCACTTGTTCTTTAAATGAGGACTAGTATGAATGGCATCACGAGGGTTACACTGTCTCCCACCTCTAATCAATGAAACTGATCCCCCCGTGAAGAAGCGGGGATAAGAATATAAGACGAGAAGACCCCATGGAGCTTTAAACTCAGTATCAACCACCAAGACTCATAACATAAAAATAGGGGGCCCTGATTACTAGTTTTTGGTTGGGGTGACCGCGGAGTAAAATGAAACCTCCACAATGAATGACACCTCCCCTCTTATCCAAGAGCCTACAACTCTAAGAATCAATATATTGACATCCATTGACCCAAACAACTTTGATCAACGAACCAAGTTACCCTGGGGATAACAGCGCAATCCATTTCAAGAGCCCCTATCGACAAATGGGTTTACGACCTCGATGTTGGATCAGGGTGTCCCAGTGGTGCAGCCGCTACTAAAGGTTCGTTTGTTCAACGATTAAAACCCTACGTGATCTGAGTTCAGACCGGA

>Seq1 [organism-Leptodactylus mystaceus] Leptodactylus mystaceus 16S ribosomal RNA, partial cds; mitochondrial.

CCGCGGTATCCTAACCGTGCGAAGGTAGCGTAATCACTTGTTCTTTAAATGAGGACTAGTATGAATGGCACCACGAAGGTTATACTGTCTCCTTTTTCTAATCAGTGAAACTAATCTTCCCGTGAAGAAGCGGGAATAAAAATATAAGACGAGAAGACCCTATGGAGCTTTAAACAAATATACACCTGCCTTCCAATCAAAAAATTTCAGTAGACAAATAATTACCCAGGCATTTTGATATAGCGTTTTAGGTTGGGGTGACCGCGGAGCAAAAAACAACCTCCGCAACGAACGGGAAATCTTTCCCTAAACCAAGGGCCACGACCCCAAGTATCGATAAATCGACACTAATTGACCCAATAAACTTGATCAATGAACCAAGTTACCCTAGGGATAACAGCGCAATCCACTTCAAGAGCCCCTATCGACAAGTGGGTTTACGACCTCGATGTTGGATCAGGGTATCCCAGTGGTGCAGCCGCTACTAACGGTTCGTTTGTTCAACGATTAAAACCCTACGTGATCTGAGTTCAGACCGGA

>Seq2 [organism-Leptodactylus mystaceus] Leptodactylus mystaceus 16S ribosomal RNA, partial cds; mitochondrial.

CCGCGGTATCCTAACCGTGCGAAGGTAGCGTAATCACTTGTTCTTTAAATGAGGACTAGTATGAATGGCACCACGAAGGTTATACTGTCTCCTTTTTCTAATCAGTGAAACTAATCTTCCCGTGAAGAAGCGGGAATAAAAATATAAGACGAGAAGACCCTATGGAGCTTTAAACAAATATACACCTGCCTTCCAATCAAAAAATTTCAGTAGACAAATAATTACCCAGGCATTTTGATATAGCGTTTTAGGTTGGGGTGACCGCGGAGCAAAAAACAACCTCCGCAACGAACGGGAAATCTTTCCCTAAACCAAGGGCCACGACCCCAAGTATCGATAAATCGACACTAATTGACCCAATAAACTTGATCAATGAACCAAGTTACCCTAGGGATAACAGCGCAATCCACTTCAAGAGCCCCTATCGACAAGTGGGTTTACGACCTCGATGTTGGATCAGGGTATCCCAGTGGTGCAGCCGCTACTAACGGTTCGTTTGTTCAACGATTAAAACCCTACGTGATCTGAGTCAAGACCGGA

>Seq3 [organism-Leptodactylus mystaceus] Leptodactylus mystaceus 16S ribosomal RNA, partial cds; mitochondrial.

CCGCGGTATCCTAACCGTGCGAAGGTAGCGTAATCACTTGTTCTTTAAATGAGGACTAGTATGAATGGCACCACGAAGGTTATACTGTCTCCTTTTTCTAATCAGTGAAACTAATCTTCCCGTGAAGAAGCGGGAATAAAAATATAAGACGAGAAGACCCTATGGAGCTTTAAACAAATATACACCTGCCTTCCAATCAAAAAATTTCAGTAGACAAATAATTACCCAGGCATTTTGATATAGCGTTTTAGGTTGGGGTGACCGCGGAGCAAAAAACAACCTCCGCAACGAACGGGAAATCTTTCCCTAAACCAAGGGCCACGACCCCAAGTATCGATAAATCGACACTAATTGACCCAATAAACTTGATCAATGAACCAAGTTACCCTAGGGATAACAGCGCAATCCACTTCAAGAGCCCCTATCGACAAGTGGGTTTACGACCTCGATGTTGGATCAGGGTATCCCAGTGGTGCAGCCGCTACTAACGGTTCGTTTGTTCAACGATTAAAACCCTACGTGATCTGAGTCCAGACCGGA

>Seq1 [organism-Leptodactylus natalensis] Leptodactylus natalensis 16S ribosomal RNA, partial cds; mitochondrial.

CCGCGGTATCCTAACCGTGCGAAGGTAGCGTAATCACTTGTTCTTTAAATGAGGACTAGTATGAACGGCATCACGAGGGTTATACTGTCTCCTCCCTTTAATCAGTGAAACTGATCTTCCCGTGAAGAAGCGGGAATAAATTTATAAGACGAGAAGACCCTATGGAGCTTTAAACACACTACAATTGCCTCACTCCATGTCAATCTCTGGAAAACCGCTTATCCGGGCACTTTGATAGCAAGTTTTAGGTTGGGGTGACCGCGGAGCAAAAAACAACCTCCACAGCGAACAGGGCTTTCCCTTAAACTCAGGATTACGACCCTAAGAATCAATAAATTGACACCCATTGACCCAATTTTTTTGACCAACGAACCAAGTTACCCTAGGGATAACAGCGCAATCTACTTCAAGAGCTCCTATCGACAAGTGGGTTTACGACCTCGATGTTGGATCAGGGTACCCCAGTGGTGCAGCCGCTGCTAACGGTTCGTTTGTTCAACGATTAAAACCCTACGTGATCTGAGTTCAGACCGGA

>Seq2 [organism-Leptodactylus natalensis] Leptodactylus natalensis 16S ribosomal RNA, partial cds; mitochondrial.

CCGCGGTATCCTAACCGTGCGAAGGTAGCGTAATCACTTGTTCTTTAAATGAGGACTAGTATGAACGGCATCACGAGGGTTATACTGTCTCCTCCCTTTAATCAGTGAAACTGATCTTCCCGTGAAGAAGCGGGAATAAATTTATAAGACGAGAAGACCCTATGGAGCTTTAAACACACTACAATTGCCTCACTCCATGTCAATCTCTGGAAAACCGCTTATCCGGGCACTTTGATAGCAAGTTTTAGGTTGGGGTGACCGCGGAGCAAAAAACAACCTCCACAGCGAACAGGGCTTTCCCTTAAACTCAGGATTACGACCCTAAGAATCAATAAATTGACACCCATTGACCCAATTTTTTTGACCAACGAACCAAGTTACCCTAGGGATAACAGCGCAATCTACTTCAAGAGCTCCTATCGACAAGTGGGTTTACGACCTCGATGTTGGATCAGGGTACCCCAGTGGTGCAGCCGCTGCTAACGGTTCGTTTGTTCAACGATTAAAACCCTACGTGATCTGAGTTCAGACCGGA

>Seq3 [organism-Leptodactylus natalensis] Leptodactylus natalensis 16S ribosomal RNA, partial cds; mitochondrial.

CCGCGGTATCCTAACCGTGCGAAGGTAGCGTAATCACTTGTTCTTTAAATGAGGACTAGTATGAACGGCATCACGAGGGTTATACTGTCTCCTCCCTTTAATCAGTGAAACTGATCTTCCCGTGAAGAAGCGGGAATAAATCTATAAGACGAGAAGACCCTATGGAGCTTTAAACACACTACAATTGCCTCACTCTATGTCAATCTCTGGAAAACCGCTTATCCGGGCACTTTGATAGCAAGTTTTAGGTTGGGGTGACCGCGGAGCAAAAAACAACCTCCACAGCGAACAGGGCTTTCCCTTAAACTCAGGATTACGACCCTAAGAATCAATAAATTGACACCCATTGACCCAATTTTTTTGACCAACGAACCAAGTTACCCTAGGGATAACAGCGCAATCTACTTCAAGAGCTCCTATCGACAAGTGGGTTTACGACCTCGATGTTGGATCAGGGTACCCCAGTGGTGCAGCCGCTGCTAACGGTTCGTTTGTTCAACGATTAAAACCCTACGTGATCTGAGTTCAGACCGGA

>Seq4 [organism-Leptodactylus natalensis] Leptodactylus natalensis 16S ribosomal RNA, partial cds; mitochondrial.

CCGCGGTATCCTAACCGTGCGAAGGTAGCGTAATCACTTGTTCTTTAAATGAGGACTAGTATGAACGGCATCACGAGGGTTATACTGTCTCCTCCCTTTAATCAGTGAAACTGATCTTCCCGTGAAGAAGCGGGAATAAATCTATAAGACGAGAAGACCCTATGGAGCTTTAAACACACTACAATTGCCTCACTCTATGTCAATCTCTGGAAAACCGCTTATCCGGGCACTTTGATAGCAAGTTTTAGGTTGGGGTGACCGCGGAGCAAAAAACAACCTCCACAGCGAACAGGGCTTTCCCTTAAACTCAGGATTACGACCCTAAGAATCAATAAATTGACACCCATTGACCCAATTTTTTTGACCAACGAACCAAGTTACCCTAGGGATAACAGCGCAATCTACTTCAAGAGCTCCTATCGACAAGTGGGTTTACGACCTCGATGTTGGATCAGGGTACCCCAGTGGTGCAGCCGCTGCTAACGGTTCGTTTGTTCAACGATTAAAACCCTACGTGATCTGAGtCCAGACCGGA

>Seq1 [organism-Leptodactylus macrosternum] Leptodactylus macrosternum 16S ribosomal RNA, partial cds; mitochondrial.

CCGCGGTATCCTAACCGTGCGAAGGTAGCGTAATCACTTGTTCTTTAAATGAGGACTAGTATGAATGGCACCACGAAGGTTATACTGTCTCCTTTCTTTAATCAGTGAAACTAATCTCCCCGTGAAGAAGCGGGGATAAGCCTATAAGACGAGAAGACCCTATGGAGCTTTAAACATAAGTAACAACTGCCCATTAAATTTTTTAATCTCAGGAAATTAATCACAACACTTAGGCACTTTGATTACAAGTTTTAGGTTGGGGTGACCGCGGAGCAAAAAATAACCTCCGCAGTGAATGGAACTTATTTCCTAAACCCAGGGCTACGACCCTAAGAATCAATAAATTGACACTGATTGACCCAATATTTTTGATCAATGAACCAAGTTACCCTAGGGATAACAGCGCAATCCACTTCAAGAGCCCCTATCGACAAGTGGGTTTACGACCTCGATGTTGGATCAGGGTATCCCAGTGGTGCAGCCGCTACTAAAGGTTCGTTTGTTCAACGATTAAAACCCTACGTGATCTGAGTTCAGACCGGA

>Seq1 [organism-Leptodactylus fuscus] Leptodactylus fuscus 16S ribosomal RNA, partial cds; mitochondrial.

CCGCGGTATCCTAACCGTGCGAAGGTAGCGTAATCACTTGTTCTTTAAATGAGGACTAGTATGAATGGCACCACGAAGGTTATACTGTCTCCTTTTTCTAATCAGTGAAACTAATCTTCCCGTGAAGAAGCGGGGATAAAAATATAAGACGAGAAGACCCTATGGAGCTTTAAACAAATATACAATTGCCTCCTCTAGTAAAAATTTCAGAAAAGACATTTCTATTTGGGCACTTTGATATAAAGTTTTAGGTTGGGGTGACCACGGAGTAAAAAACAACCTCCGCAGTGAAAGGGGCTTTCCCCTAAGCCAAGGGCTACAACCCTAAGAATCAATAAATTGACACTAATTGACCCAATTAATTGATCAATGAACCAAGTTACCCTAGGGATAACAGCGCAATCCACTTCAAGAGCCCCTATCGACAAGTGGGTTTACGACCTCGATGTTGGATCAGGGTATCCTAGTGGTGCAGCCGCTACTAACGGTTCGTTTGTTCAACGATTAAAACCCTACGTGATCTGAGTTCAGACCGGA

>Seq1 [organism-Leptodactylus pustulatus] Leptodactylus pustulatus 16S ribosomal RNA, partial cds; mitochondrial.

CCGCGGTATCCTAACCGTGCGAAGGTAGCGTAATCACTTGTTCTTTAAATAAGGACTAGTATGAATGGCACCACGAAGGTTATACTGTCTCCTTTCTCTAATCAGTGAAACTAATCTTCCCGTGAAGAAGCGGGAATAACCCTATAAGACGAGAAGACCCTATGGAGCTTTAAACGCAGTAACAATTGCCCACCCAAAACTATTCTCCGGAGATCACAAATTATTTGGGCACCATGATTACAAGTTTTAGGTTGGGGTGACCGCGGAGCAAAAAACAACCTCCGCAGTGAACGGGAACCTCCCTAATCTCAGGACTACACTCCTAAGAATCAACATATTGACACCCATTGACCCAATATTTGATCAATGAACCAAGTTACCCTAGGGATAACAGCGCAATCCACTTCAAGAGCTCCTATCGACAAGTGGGTTTACGACCTCGATGTTGGATCAGGGTATCCTAGTGGTGCAGCCGCTACTGAAGGTTCGTTTGTTCAACGATTGAAACCCTACGTGATCTGAGTTCAGACCGGA

>Seq1 [organism-Leptodactylus troglodytes] Leptodactylus troglodytes 16S ribosomal RNA, partial cds; mitochondrial.

CCGCGGTATCCTAACCGTGCGAAGGTAGCGTAATCACTTGTTCTTTAAATGAGGACTAGTATGAATGGCATCACGAAGGTTATACTGTCTCCTTTCCCTAATCAGTGAAACTAATCTGCCCGTGAAGAAGCGGGAATAAACCTATAAGACGAGAAGACCCTATGGAGCTTTAAACAAATACACAATTGCCCTTCCTTATAATAAATCTCAGGAAAATTTCCCCCTCTACTCGGACACTTTGATGTATAGTTTTAGGTTGGGGTGACCACGGAGTAAAAACTAACCTCCGCAGTGAACGGGGCCTCCCCCTTAACTAAGAGCTACAACTCTAAGAAACAATAAATTGACACCCATTGACCCAAATATTAATTTGATCAATGAACCAAGTTACCCTAGGGATAACAGCGCAATCCACTTCAAGAGCCCCTATCGACAAGTGGGTTTACGACCTCGATGTTGGATCAGGGTATCCCAGTGGTGCAGCCGCTACTAAAGGTTCGTTTGTTCAACGATTAAAACCCTACGTGATCTGAGTTCAGACCGGA

>Seq1 [organism-Osteocephalus taurinus] Osteocephalus taurinus 16S ribosomal RNA, partial cds; mitochondrial.

CCGCGGTATCCTAACCGTGCGAAGGTAGCGTAATCACTTGTCTTTTAAATGGGGACTTGTATGAATGGCATCACGAGGGTTATACTGTCTCCTTTTTCTAATCAGTGAAACTAATCTTCCCGTGAAGAAGCGGGAATAAACATATAAGACGAGAAGACCCTATGGAGCTTTAAACTCAATAACAAATACTAACCTTACATCAACCTTCAGAGTATTAACCTTAATTTTAGTATTATGATTATTAGTTTTAGGTTGGGGTGACCGCGGAGTAAAAATTAACCTCCACATTGAACGGGACCTGTCCCTGAGCCAAGAACTACAGCTCGAGGCACCAACAAATTGACATTAATTGACCCAATATTTTGATCAACGAACCAAGTTACCCTAGGGATAACAGCGCAATCCACTTCAAGAGCTCCTATCGACAAGTGGGTTTACGACCTCGATGTTGGATCAGGGTATCCCAGTGGTGCAGCCGCTACTAAAGGTTCGTTTGTTCAACGATTAAAACCCTACGTGATCTGAGTTCAGACCGGA

>Seq2 [organism-Osteocephalus taurinus] Osteocephalus taurinus 16S ribosomal RNA, partial cds; mitochondrial.

CCGCGGTATCCTAACCGTGCGAAGGTAGCGTAATCACTTGTCTTTTAAATGGGGACTTGTATGAATGGCATCACGAGGGTTATACTGTCTCCTTTTTCTAATCAGTGAAACTAATCTTCCCGTGAAGAAGCGGGAATAAACATATAAGACGAGAAGACCCTATGGAGCTTTAAACTCAATAACAAATACTAACCTTACATCAACCTTCAGAGTATTAACCTTAATTTTAGTATTATGATTATTAGTTTTAGGTTGGGGTGACCGCGGAGTAAAAATTAACCTCCACATTGAACGGGACCTGTCCCTGAGCCAAGAACTACAGCTCGAGGCACCAACAAATTGACATTAATTGACCCAATATTTTGATCAACGAACCAAGTTACCCTAGGGATAACAGCGCAATCCACTTCAAGAGCTCCTATCGACAAGTGGGTTTACGACCTCGATGTTGGATCAGGGTATCCCAGTGGTGCAGCCGCTACTAAAGGTTCGTTTGTTCAACGATTAAAACCCTACGTGATCTGAGTTCAGACCGGA

>Seq3 [organism-Trachycephalus typhonius] Trachycephalus typhonius 16S ribosomal RNA, partial cds; mitochondrial.

ccGcGGTAtCCTAACCGtGCGAAGGTAgCgTAAtCACTTGTTCTTTAAATGAGGACTAGTATGAATGGCACCACGAGGGTTATACTGTCTCCTTTTTCTAATCAGTGAAACTAATCTCCCCGTGAAGAAGCGGGGATTAAAATATAAGACGAGAAGACCCTATGGAGCTTTAAACTTAATAACAAGTACTATCTTTTATTTAACTTTCAGAACATATACCTTTATTCTAGTATTATGATTATTAGTTTTAGGTTGGGGTGACCGCGGAGCAAAAATCAACCTCCACATTGAACGGAACTCATTCCTGAGCAAAAAGCCACGACTTTAAGCACCAACAAATTGACATCAATTGACCCAATATTTTGATCAACGAACCAAGTTACCCTAGGGATAACAGCGCAATCCACTTCAAGAGCTCCTATCGACAAGTGGGTTTACGACCTCGATGTTGGATCAGGGTATCCCAGTGGTGCAGCCGCTACTAAAGGTTCGTTTGTTCAACGATTAAAACCCTACGTGATCTGAGTTCAGACCGGA

>Seq1 [organism-Physalaemus cuvieri] Physalaemus cuvieri 16S ribosomal RNA, partial cds; mitochondrial.

CCGCGGTATCCTAACCGTGCGAAGGTAGCGTAATCACTTGTTCTTTAAATGAGGACTAGTATGAATGGCATCACGAAGGTTATACTGTCTCCCCTTTTTAATCAGTGAAACTAATCTTCCCGTGAAGAAGCGGGAATAAAATTATAAGACGAGAAGACCCTATGGAGCTTTAGACTACGCAACAATTGCTATTCACTTTTTACCCTATGGGGCCCCAACTATATTTTCTCATATTGATTGCTAGTCTTGGGTTGGGGTGACCGCGGAGTAAAATATAACCTCCACGATGAAAGAAACTTAATTTCTTAACCCAGAGTTACAACTCTAAGTACCAAAAATTTGACATTAATTGATCCAATTTATTGATCAACGAACCAAGTTACCCTAGGGATAACAGCGCAATCTACTTCAAGAGTCCCTATCGACAAGTAGGTTTACGACCTCGATGTTGGATCAGGGTATCCTAGTGGTGCAGCCGCTACTAAAGGTTCGTTTGTTCAACGATTAAAACCCTACGTGATCTGAGTTCAGACCGGA

>Seq2 [organism-Physalaemus cuvieri] Physalaemus cuvieri 16S ribosomal RNA, partial cds; mitochondrial.

CCGCGGTATCCTAACCGTGCGAAGGTAGCGTAATCACTTGTTCTTTAAATGAGGACTAGTATGAATGGCATCACGAAGGTTATACTGTCTCCCCTTTTTAATCAGTGAAACTAATCTTCCCGTGAAGAAGCGGGAATAAAATTATAAGACGAGAAGACCCTATGGAGCTTTAGACTACGCAACAATTGCTATTCACTTTTTACCCTATGGGGCCCCAACTATATTTTCTCATATTGATTGCTAGTCTTGGGTTGGGGTGACCGCGGAGTAAAATATAACCTCCACGATGAAAGAAACTTAATTTCTTAGCCCAGAGTTACAACTCTAAGTACCAAAAAATTTGACATGAATTGATCCAATTTATTGATCAACGAACCAAGTTACCCTAGGGATAACAGCGCAATCTACTTCAAGAGTCCCTATCGACAAGTAGGTTTACGACCTCGATGTTGGATCAGGGTATCCTAGTGGTGCAGCCGCTACTAAAGGTTCGTTTGTTCAACGATTAAAACCCTACGTGATCTGAGTTCAGACCGGA

>Seq3 [organism-Physalaemus cuvieri] Physalaemus cuvieri 16S ribosomal RNA, partial cds; mitochondrial.

CCGCGGTATCCTAACCGTGCGAAGGTAGCGTAATCACTTGTTCTTTAAATGAGGACTAGTATGAATGGCATCACGAAGGTTATACTGTCTCCCCTTTTTAATCAGTGAAACTAATCTTCCCGTGAAGAAGCGGGAATAAAATTATAAGACGAGAAGACCCTATGGAGCTTTAGACTACGCAACAATTGCTATTCACTTTTTACCCTATGGGGCCCCAACTATATTTTCTCATATTGATTGCTAGTCTTGGGTTGGGGTGACCGCGGAGTAAAATATAACCTCCACGATGAAAGAAACTTAATTTCTTAACCCAGAGTTACAACTCTAAGTACCAAAAATTTGACATTAATTGACCCAATTTATTGATCAACGAACCAAGTTACCCTAGGGATAACAGCGCAATCTACTTCAAGAGTCCCTATCGACAAGTAGGTTTACGACCTCGATGTTGGATCAGGGTATCCTAGTGGTGCAGCCGCTACTAAAGGTTCGTTTGTTCAACGATTAAAACCCTACGTGATCTGAGTTCAGACCGGA

>Seq4 [organism-Physalaemus cuvieri] Physalaemus cuvieri 16S ribosomal RNA, partial cds; mitochondrial.

CCGCGGTATCCTAACCGTGCGAAGGTAGCGTAATCACTTGTTCTTTAAATGAGGACTAGTATGAATGGCATCACGAAGGTTATACTGTCTCCCCTTTTTAATCAGTGAAACTAATCTTCCCGTGAAGAAGCGGGAATAAAATTATAAGACGAGAAGACCCTATGGAGCTTTAGACTACGCAACAATTGCTATTCACTTTTTACCCCATGGAGCCCCAACCATATTTTCTCATATTGATTGCTAGTCTTGGGTTGGGGTGACCGCGGAGTAAAATATAACCTCCACGATGAAAGAAACTTAATTTCTTAACCCAGAGTTACAACTCTAAGTACCAAAAATTTGACATTAATTGATCCAATTTATTGATCAACGAACCAAGTTACCCTAGGGATAACAGCGCAATCTACTTCAAGAGTCCCTATCGACAAGTAGGTTTACGACCTCGATGTTGGATCAGGGTATCCTAGTGGTGCAGCCGCTACTAAAGGTTCGTTTGTTCAACGATTAAAACCCTACGTGATCTGAGTTCAGACCGGA

>Seq5 [organism-Physalaemus cuvieri] Physalaemus cuvieri 16S ribosomal RNA, partial cds; mitochondrial.

CCGCGGTATCCTAACCGTGCGAAGGTAGCGTAATCACTTGTTCTTTAAATGAGGACTAGTATGAATGGCATCACGAAGGTTATACTGTCTCCCCTTTTTAATCAGTGAAACTAATCTTCCCGTGAAGAAGCGGGAATAAAATTATAAGACGAGAAGACCCTATGGAGCTTTAGACTACGCAACAATTGCTATTCACTTTTTACCCTATGGGGCCCCAACTATATTTTCTCATATTGATTGCTAGTCTTGGGTTGGGGTGACCGCGGAGTAAAATATAACCTCCACGATGAAAGAAACTTAATTTCTTAACCCAGAGTTACAACTCTAAGTACCAAAAATTTGACATTAATTGATCCAATTTATTGATCAACGAACCAAGTTACCCTAGGGATAACAGCGCAATCTACTTCAAGAGTCCCTATCGACAAGTAGGTTTACGACCTCGATGTTGGATCAGGGTATCCTAGTGGTGCAGCCGCTACTAAAGGTTCGTTTGTTCAACGATTAAAACCCTACGTGATCTGAGTTCAGACCGGA

>Seq6 [organism-Physalaemus cuvieri] Physalaemus cuvieri 16S ribosomal RNA, partial cds; mitochondrial.

CCGCGGTATCCTAACCGTGCGAAGGTAGCGTAATCACTTGTTCTTTAAATGAGGACTAGTATGAATGGCATCACGAAGGTTATACTGTCTCCCCTTTTTAATCAGTGAAACTAATCTTCCCGTGAAGAAGCGGGAATAAAATTATAAGACGAGAAGACCCTATGGAGCTTTAGACTACGCAACAATTGCTATTCACTTTTTACCCTATGGGGCCCCAACTATATTTTCTCATATTGATTGCTAGTCTTGGGTTGGGGTGACCGCGGAGTAAAATATAACCTCCACGATGAAAGAAACTTAATTTCTTAACCCAGAGTTACAACTCTAAGTACCAAAAAATTTGACATGAATTGATCCAATTTATTGATCAACGAACCAAGTTACCCTAGGGATAACAGCGCAATCTACTTCAAGAGTCCCTATCGACAAGTAGGTTTACGACCTCGATGTTGGATCAGGGTATCCTAGTGGTGCAGCCGCTACTAAAGGTTCGTTTGTTCAACGATTAAAACCCTACGTGATCTGAGTTCAGACCGGA

>Seq1 [organism-Physalaemus nattereri] Physalaemus nattereri 16S ribosomal RNA, partial cds; mitochondrial.

CCGCGGTATCCTAACCGTGCGAAGGTAGCGTAATCACTTGTTCTTTAAATGAGGACTAGTATGAATGGCATCACGAGGGTTAAACTGTCTCCCCTCTCTAATCAGTGAAACTGATCTCCCCGTGAAGAAGCGGGGATATTTTTATAAGACGAGAAGACCCTATGGAGCTTTAAACCGCGCAACAACTGCTTTCCCACCTTATACTTCCAGGATAATAAACTATATCTTAGCATAATGTTAACCGGTTTTCGGTTGGGGTGACCGCGGAGCAAAAATTAACCTCCACGATGAAAGAAACTTTATTTCTTAACCCAGAGCTACAACTCAAAGTATCAAAAACCTGACATAAATTGATCCAATCTATTTGATCAACGAACCAAGTTACCCTAGGGATAACAGCGCAATCTACTTCAAGAGCCCCTATCGACAAGTGGGTTTACGACCTCGATGTTGGATCAGGGTATCCTAGTGGTGCAGCCGCTACTAAAGGTTCGTTTGTTCAACGATTAAAACCCTACGTGATCTGAGTCAAGACCGGA

>Seq2 [organism-Physalaemus nattereri] Physalaemus nattereri 16S ribosomal RNA, partial cds; mitochondrial.

CCGCGGTATCCTAACCGTGCGAAGGTAGCGTAATCACTTGTTCTTTAAATGAGGACTAGTATGAATGGCATCACGAGGGTTAAACTGTCTCCCCTCTCTAATCAGTGAAACTGATCTCCCCGTGAAGAAGCGGGGATATTTTTATAAGACGAGAAGACCCTATGGAGCTTTAAACCGCGCAACAACTGCTTTCCCACCTTATACTTCCAGGATAATAAACTATATCTTAGCATAATGTTAACCGGTTTTCGGTTGGGGTGACCGCGGAGCAAAAATTAACCTCCACGATGAAAGAAACTTTATTTCTTAACCCAGAGCTACAACTCAAAGTATCAAAAACCTGACATAAATTGATCCAATCTATTTGATCAACGAACCAAGTTACCCTAGGGATAACAGCGCAATCTACTTCAAGAGCCCCTATCGACAAGTGGGTTTACGACCTCGATGTTGGATCACGGTATCCCAGTGGTGCAGCCGCTACTAAAGGTTCGTTTGTTCAACGATTAAAACCCTACGTGATCTGAGTTCAGACCGGA

>Seq3 [organism-Physalaemus nattereri] Physalaemus nattereri16S ribosomal RNA, partial cds; mitochondrial.

CCGCGGTATCCTAACCGTGCGAAGGTAGCGTAATCACTTGTTCTTTAAATGAGGACTAGTATGAATGGCATCACGAGGGTTAAACTGTCTCCCCTCTCTAATCAGTGAAACTGATCTCCCCGTGAAGAAGCGGGGATATTTTTATAAGACGAGAAGACCCTATGGAGCTTTAAACCGCGCAACAACTGCTTTCCCACCTTATACTTCCAGGATAATAAACTATATCTTAGCATAATGTTAACCGGTTTTCGGTTGGGGTGACCGCGGAGCAAAAATTAACCTCCACGATGAAAGAAACTTTATTTCTTAACCCAGAGCTACAACTCAAAGTATCAAAAACCTGACATAAATTGATCCAATCTATTTGATCAACGAACCAAGTTACCCTAGGGATAACAGCGCAATCTACTTCAAGAGCCCCTATCGACAAGTGGGTTTACGACCTCGATGTTGGATCAGGGTATCCTAGTGGTGCAGCCGCTACTAAAGGTTCGTTTGTTCAACGATTAAAACCCTACGTGATCTGAGTTCAGACCGGA

>Seq4 [organism-Physalaemus nattereri] Physalaemus nattereri 16S ribosomal RNA, partial cds; mitochondrial.

CCGCGGTATCCTAACCGTGCGAAGGTagCGTAATCACTTGTTCTTTAAATGAGGACTAGTATGAATGGCATCACGAGGGTTAAACTGTCTCCCCTCTCTAATCAGTGAAACTGATCTCCCCGTGAAGAAGCGGGGATATTTTTATAAGACGAGAAGACCCTATGGAGCTTTAAACCGCGCAACAACTGCTTTCCCACCTTATACTTCCAGGATAATAAACTATATCTTAGCATAATGTTAACCGGTTTTCGGTTGGGGTGACCGCGGAGCAAAAATTAACCTCCACGATGAAAGAAACTTTATTTCTTAACCCAGAGCTACAACTCAAAGTATCAAAAACCTGACATAAATTGATCCAATCTATTTGATCAACGAACCAAGTTACCCTAGGGATAACAGCGCAATCTACTTCAAGAGCCCCTATCGACAAGTGGGTTTACGACCTCGATGTTGGATCAGGGTATCCTAGTGGTGCAGCCGCTACTAAAGGTTCGTTTGTTCAACGATTAAAACCCTACGTGATCTGAGTTCAGACCGGA

>Seq5 [organism-Physalaemus nattereri] Physalaemus nattereri 16S ribosomal RNA, partial cds; mitochondrial.

CCGCGGTATCCTAACCGTGCGAAGGTAGCGTAATCACTTGTTCTTTAAATGAGGACTAGTATGAATGGCATCACGAGGGTTAAACTGTCTCCCCTCTCTAATCAGTGAAACTGATCTCCCCGTGAAGAAGCGGGGATATTTTTATAAGACGAGAAGACCCTATGGAGCTTTAAACCGCGCAACAACTGCTTTCCCACCTTATACTTCCAGGATAATAAACTATATCTTAGCATAATGTTAACCGGTTTTCGGTTGGGGTGACCGCGGAGCAAAAATTAACCTCCACGATGAAAGAAACTTTATTTCTTAACCCAGAGCTACAACTCAAAGTATCAAAAACCTGACATAAATTGATCCAATCTATTTGATCAACGAACCAAGTTACCCTAGGGATAACAGCGCAATCTACTTCAAGAGCCCCTATCGACAAGTGGGTTTACGACCTCGATGTTGGATCAGGGTATCCTAGTGGTGCAGCCGCTACTAAAGGTTCGTTTGTTCAACGATTAAAACCCTACGTGATCTGAGTTCAGACCGGA

>Seq1 [organism-Rhinella mirandaribeiroi] Rhinella mirandaribeiroi 16S ribosomal RNA, partial cds; mitochondrial.

CCGCGGTATCCTAACCGTGCGAAGGTAGCGTAATCACTTGTTCTTTAAATGTGGACTAGTATGAACGGCACCACGAAGGTTATACTGTCTCCTTTTTCTAATCAGTGAAACTAATCTCCCCGTGAAGAAGCGGGGATAATTCTATAAGACGAGAAGACCCTATGGAGCTTTAAACAACACAGCACTTACTAAATATCCTCTAATGTTCCTGAAATATTAACTCTTTAAAGTAAGATGACTGCAAGTTTTCGGTTGGGGTGACCACGGAGTAAATCATAACCTCCATGTTGAAAGAACTTTTCTAAGCCAAGAATAACCAATCTAAGCATTAACAAATTAACATCTATTGACCCAATAAATTTGATCAACGAACCAAGTTACCCTAGGGATAACAGCGCAATCCACTTCAAGAGCTCCTATCGACAAGTGGGTTTACGACCTCGATGTTGGATCAGGGTATCCCAGTGGTGCAGCCGCTACTAAAGGTTCGTTTGTTCAACGATTAAAACCCTACGTGATCTGAGTTCAGACCGGA

>Seq2 [organism-Rhinella mirandaribeiroi] Rhinella mirandaribeiroi 16S ribosomal RNA, partial cds; mitochondrial.

CCGCGGTATCCTAACCGTGCGAAGGTAGCGTAATCACTTGTTCTTTAAATGTGGACTAGTATGAACGGCACCACGAAGGTTATACTGTCTCCTTTTTCTAATCAGTGAAACTAATCTCCCCGTGAAGAAGCGGGGATAATTCTATAAGACGAGAAGACCCTATGGAGCTTTAAACAACACAGCACTTACTAAATATCCTCTAATGTTCCTGAAATATTAACTCTTTAAAGTAAGATGACTGCAAGTTTTCGGTTGGGGTGACCACGGAGTAAATCATAACCTCCATGTTGAAAGAACTTTTCTAAGCCAAGAATAACCAATCTAAGCATTAACAAATTAACATCTATTGACCCAATAAATTTGATCAACGAACCAAGTTACCCTAGGGATAACAGCGCAATCCACTTCAAGAGCTCCTATCGACAAGTGGGTTTACGACCTCGATGTTGGATCAGGGTATCCCAGTGGTGCAGCCGCTACTAAAGGTTCGTTTGTTCAACGATTAAAACCCTACGTGATCTGAGTTCAGACCGGA

>Seq3 [organism-Rhinella mirandaribeiroi] Rhinella mirandaribeiroi 16S ribosomal RNA, partial cds; mitochondrial.

CCGCGGTATCCTAACCGTGCGAAGGTAGCGTAATCACTTGTTCTTTAAATGTGGACTAGTATGAACGGCACCACGAAGGTTATACTGTCTCCTTTTTCTAATCAGTGAAACTAATCTCCCCGTGAAGAAGCGGGGATAATTCTATAAGACGAGAAGACCCTATGGAGCTTTAAACAACACAGCACTTACTAAATATCCTCTAATGTTCCTGAAATATTAACTCTTTAAAGTAAGATGACTGCAAGTTTTCGGTTGGGGTGACCACGGAGTAAATCATAACCTCCATGTTGAAAGAACTTTTCTAAGCCAAGAATAACCAATCTAAGCATTAACAAATTAACATCTATTGACCCAATAAATTTGATCAACGAACCAAGTTACCCTAGGGATAACAGCGCAATCCACTTCAAGAGCTCCTATCGACAAGTGGGTTTACGACCTCGATGTTGGATCAGGGTATCCCAGTGGTGCAGCCGCTACTAAAGGTTCGTTTGTTCAACGATTAAAACCCTACGTGATCTGAGTCCAGACCGGa

>Seq4 [organism-Rhinella diptycha] Rhinella diptycha 16S ribosomal RNA, partial cds; mitochondrial.

CCGCGGTATCCTAACCGTGCGAAGGTAGCGTAATCACTTGTTCTTTAAATGTGGACTAGTATGAATGGCACCACGAAGGTTATACTGTCTCCTTTTTCTAATCAGTGAAACTAATCTCCCCGTGAAGAAGCGAGGATACACCTATAAGACGAGAAGACCCTATGGAGCTTTAAACAACACAGCAATTATCTGCTAAACTAAGAAGTTTCTGAACATTTTTAATCTTTTAAGTAATCTGACTGCAAGTTTTTGGTTGGGGTGACCACGGAGCATAACATAACCTCCATGTTGAAAGAATTCTTTCTAAGCCAAGAACAACCTTTCTAAGCATCAATATATTGACATCCATTGACCCAATACATTTGACCAACGAACCAAGTTACCCTAGGGATAACAGCGCAATCCACTTCAAGAGCTCCTATCGACAAGTGGGTTTACGACCTCGATGTTGGATCAGGGTATCCCAGTGGTGCAGCCGCTACTAAAGGTTCGTTTGTTCAACGATTAAAACCCTACGTGATCTGAGTTCAGACCGGA

>Seq1 [organism-Scinax fuscomarginatus] Scinax fuscomarginatus 16S ribosomal RNA, partial cds; mitochondrial.

CCGcGGTATCCTAACCGTGCGAAGGTAGCGTAATCACTTGTTCTTTAAATGGGGACTTGTATGAATGGCATCACGAGGGTTATACTGTCTCCTTTTTCCAATCAGTGAATCTAATCTCCCCGTGAAGAAACGGGGATACAACTATAAGACGAGAAGACCCTATGGAGCTTTAAATACCTCTTCAAACGTTGGACCTTTCCCCCCCCCAGTTTTTAACTCACTTATATTAACCCTGAACGCCATGAAGACAACTTTTAGGTTGGGGTGACCGCGGAGAACAACAAAACCTCCACGCTGAATCAGACTTTCCAAGCCTGAACTAAGAGCCACCGCTCAATGCACTAAAAAATTAACATCTATTGACCCAATACATCACTTGATCAACGAACCAAGTTACCCTAGGGATAACAGCGCAATCCATTTCAAGAGTCCATATCGACAAGTGGGTTTACGACCTCGATGTTGGATCAGGGTATCCCAGTGGTGCAGCCGCTACTAATGGTTCGTTTGTTCAACGATTAAAACCCTACGTGATCTGAGTTCAGACCGGA

>Seq2 [organism-Scinax fuscomarginatus] Scinax fuscomarginatus 16S ribosomal RNA, partial cds; mitochondrial.

CCGCGGTATCCTAACCGTGCGAAGGTAGCGTAATCACTTGTTCTTTAAATGGGGACTTGTATGAATGGCATCACGAGGGTTATACTGTCTCCTTTTTCCAATCAGTGAATCTAATCTCCCCGTGAAGAAACGGGGATACAACTATAAGACGAGAAGACCCTATGGAGCTTTAAATACCTCTTCAAACGTTGGACCTTTCCCCCCAGTTTTTAACTCACTTATATTAACCCTGAACGCCATGAAGACAACTTTTAGGTTGGGGTGACCGCGGAGAACAACAAAACCTCCACGCTGAATCAGACTTTCCAAGCCTGAACTAAGAGCCACCGCTCAATGCACTAAAAAATTAACATCTATTGACCCAATACATCACTTGATCAACGAACCAAGTTACCCTAGGGATAACAGCGCAATCCATTTCAAGAGTCCATATCGACAAGTGGGTTTACGACCTCGATGTTGGATCAGGGTATCCCAGTGGTGCAGCCGCTACTAATGGTTCGTTTGTTCAACGATTAAAACCCTACGTGATCTGAGTTCAGACCGGA

>Seq3 [organism-Scinax fuscomarginatus] Scinax fuscomarginatus 16S ribosomal RNA, partial cds; mitochondrial.

CCGCGGTATCCTAACCGTGCGAAGGTAGCGTAATCACTTGTTCTTTAAATGGGGACTTGTATGAATGGCATCACGAGGGTTATACTGTCTCCTTTTTCCAATCAGTGAATCTAATCTCCCCGTGAAGAAACGGGGATACAACTATAAGACGAGAAGACCCTATGGAGCTTTAAATACCTCTTCAAACGTTGGACCTTTCCCCCCAGTTTTTAACTCACTTATATTAACCCTGAACGCCATGAAGACAACTTTTAGGTTGGGGTGACCGCGGAGAACAACAAAACCTCCACGCTGAATCAGACTTTCCAAGCCTGAACTAAGAGCCACCGCTCAATGCACTAAAAAATTAACATCTATTGACCCAATACATCACTTGATCAACGAACCAAGTTACCCTAGGGATAACAGCGCAATCCATTTCAAGAGTCCATATCGACAAGTGGGTTTACGACCTCGATGTTGGATCAGGGTATCCCAGTGGTGCAGCCGCTACTAATGGTTCGTTTGTTCAACGATTAAAACCCTACGTGATCTGAGTTCAGACCGGA

>Seq1 [organism-Scinax cf. similis] Scinax cf. similis 16S ribosomal RNA, partial cds; mitochondrial.

CCGCGGTATCCTAACCGTGCGAAGGTAGCGTAATCACTTGTTCTTTAAATGTGGACTAGTATGAACGGCATCACGAGGGTTATACTGTCTCCTTTCTCTAATCAGTGAAACTAATCCCCCCGTGAAGAAGCGGGGATAAAACTATAAGACGAGAAGACCCTATGGAGCTTTAAAGTTCTTTTCAAACGCCAAACCTCTTCCAAGTCACAGACTCCTGCACGAAACCACGCGCCATGAAGACAACTTTTAGGTTGGGGTGACCATGGAGTATAAGACAACCTCCACGCTGAACAGGCCTCTAAACCTTGAGCCAAGGACCACAGCCCGCCGCACTAAAAAATTAACACACATTGATCCAATGAATTTTGATCAACGAACCAAGTTACCCTAGGGATAACAGCGCAATCTACTTCAAGAGTTCCTATCGACAAGTGGGTTTACGACCTCGATGTTGGATCAGGGTGTCCCAGTGGTGCAGCCGCTACTAATGGTTCGTTTGTTCAACGATTAAAACCCTACGTGATCTGAGTTCAGACCGGA

>Seq2 [organism-Scinax cf. similis] Scinax cf. similis 16S ribosomal RNA, partial cds; mitochondrial.

CCGCGGTATCCTAACCGTGCGAAGGTAGCGTAATCACTTGTTCTTTAAATGTGGACTAGTATGAACGGCATCACGAGGGTTATACTGTCTCCTTTCTCTAATCAGTGAAACTAATCCCCCCGTGAAGAAGCGGGGATAAAACTATAAGACGAGAAGACCCTATGGAGCTTTAAAGTTCTTTTCAAACGCCAAACCTCTTCCAAGTCACAGACTCCTGCACGAAACCACGCGCCATGAAGACAACTTTTAGGTTGGGGTGACCATGGAGTATAAGACAACCTCCACGCTGAACAGGCCTCTAAACCTTGAGCCAAGGACCACAGCCCGCCGCACTAAAAAATTAACACACATTGATCCAATGAATTTTGATCAACGAACCAAGTTACCCTAGGGATAACAGCGCAATCTACTTCAAGAGTTCCTATCGACAAGTGGGTTTACGACCTCGATGTTGGATCAGGGTGTCCCAGTGGTGCAGCCGCTACTAATGGTTCGTTTGTTCAACGATTAAAACCCTACGTGATCTGAGTTCAGACCGGA

>Seq1 [organism-Scinax cf. nebulosus] Scinax cf. nebulosus 16S ribosomal RNA, partial cds; mitochondrial.

CCGCGGTATCCTAACCGTGCGAAGGTAGCGTAATCACTTGTTCTTTAAATGGGGACTAGTATGAATGGCACCACGAGGGTTATACTGTCTCCCTTTTCCAATCAGTGAAACTAATCTCCCTGTGAAGAAGCAGGGATAAAAATATAAGACGAGAAGACCCTATGGAGCTTTAAATTTCAATTCAAATGCCCTCCAACCCTAAAAGTTTCCAACTTTATTCTCACAAACCAGGCATAATGAAATAGAATTTTAGGTTGGGGTGACCGCGGAGTACAAAACAACCTCCACATTGAACTAGGCCTATTACCTTGAGCAACGAACTACAATCCTATGCACAAAAAAATTTACATTCATTGACCCAATTTTTTTGATCAACGAACCAAGTTACCCTAGGGATAACAGCGCAATCCTCTTCAAGAGCCCCTATCGCCAAGAGGGTTTACGACCTCGATGTTGGATCAGGGTATCCCAGTGGCGCAGCCGCTACTAATGGTTCGTTTGTTCAACGATTAAAACCCTACGTGATCTGAGTTCAGACCGGA

>Seq2 [organism-Scinax cf. nebulosus] Scinax cf. nebulosus 16S ribosomal RNA, partial cds; mitochondrial.

CCGCGGTATCCTAACCGTGCGAAGGTAGCGTAATCACTTGTTCTTTAAATGGGGACTAGTATGAATGGCACCACGAGGGTTATACTGTCTCCCTTTTCCAATCAGTGAAACTAATCTCCCTGTGAAGAAGCAGGGATAAAAATATAAGACGAGAAGACCCTATGGAGCTTTAAATTTCAATTCAAATGCCCTCCAACCCTAAAAGTTTCCAACTTTATTCTCACAAACCAGGCATAATGAAATAGAATTTTAGGTTGGGGTGACCGCGGAGTACAAAACAACCTCCACATTGAACTAGGCCTATTACCTTGAGCAACGAACTACAATCCTATGCACAAAAAAATTTACATTCATTGACCCAATTTTTTTGATCAACGAACCAAGTTACCCTAGGGATAACAGCGCAATCCTCTTCAAGAGCCCCTATCGCCAAGAGGGTTTACGACCTCGATGTTGGATCAGGGTATCCCAGTGGCGCAGCCGCTACTAATGGTTCGTTTGTTCAACGATTAAAACCCTACGTGATCTGAGTTCAGACCGGA

>Seq1 [organism-Scinax x-sigantus] Scinax x-sigantus 16S ribosomal RNA, partial cds; mitochondrial.

CCGCGGTATCCTAACCGTGCGAAGGTAGCGTAATCACTTGTTCTTTAAATGAGGACTAGTATGAATGGCACCACGAGGGTTGCACTGTCTCCTTCCCCTAATCAATGAAACTAATCTTCCCGTGAAGAAGCGGGAGTTTATTTATAAGACGAGAAGACCCTATGGAGCTTTAAATTTCTCTTCAAACGCCAGACATAAACCCTAAACATCTTACACCAACCCGGCGCCATGAAGATGAATTTTAGGTTGGGGTGACCACGGAGAACAAGACAACCTCCATGCTGAATAGGCCTTGAAACCTTGAACTAAGGACCACAGCCCGACGAACTAAAAAATTAACACCCATTGACCCAATTCTTTTTGATCAACGAACCAAGTTACCCTAGGGATAACAGCGCAATCTACTTCAAGAGTCCTTATCGACAAGTGGGTTTACGACCTCGATGTTGGATCAGGGTACCCCAGTGGTGCAGCCGCTACTAAAGGTTCGTTTGTTCAACGATTAAAACCCTACGTGATCTGAGTTCAGACCGGA

>Seq2 [organism-Scinax x-sigantus] Scinax x-sigantus 16S ribosomal RNA, partial cds; mitochondrial.

CCGCGGTATCCTAACCGTGCGAAGGTAGCgtAATcaCTTGTTCTTTAAATGAGGACTAGTATGAATGGCAtcACGAGGGTTGCACTGTCTCCTTCCCCTAATCAATGAAACTAATCTTCCCGTGAAGAAGCGGGAATTTATTTATAAGACGAGAAGACCCTATGGAGCTTTAAATTTCTCTTCAAACGCCAGACATAAACCCTAAACATCTTACACCAACCCGGCGCCATGAAGATGAATTTTAGGTTGGGGTGACCACGGAGAACAAGACAACCTCCATGCTGAATAGGCCTAAAACCTTGAACTAAGGACCACAGCCCGACGAACTAAAAAATTAACACCCATTGACCCAATTCTTTTTGATCAACGAACCAAGTTACCCTAGGGATAACAGCGCAATCTACTTCAAGAGTCCTTATCGACAAGTGGGTTTACGACCTCGATGTTGGATCAGGGTACCCCAGTGGTGCAGCCGCTACTAAAGGGTCGTTTGTTCAACGATTAAAACCCTACGTGATCTGAGTTCAAACCGGA

>Seq3 [organism-Scinax x-sigantus] Scinax x-sigantus 16S ribosomal RNA, partial cds; mitochondrial.

CCGCGGTATCCTAACCGTGCGAAGGTAGCGTAATCACTTGTTCTTTAAATGAGGACTAGTATGAATGGCACCACGAGGGTTGCACTGTCTCCTTCCCCTAATCAATGAAACTAATCTTCCCGTGAAGAAGCGGGAATTTATTTATAAGACGAGAAGACCCTATGGAGCTTTAAATTTCTCTTCAAACGCCAGACATAAACCCTAAACATCTTACACCAACCCGGCGCCATGAAGATGAATTTTAGGTTGGGGTGACCACGGAGAACAAGACAACCTCCATGCTGAATAGGCCTAAAACCTTGAACTAAGGACCACAGCCCGACGAACTAAAAAATTAACACCCATTGACCCAATTCTTTTTGATCAACGAACCAAGTTACCCTAGGGATAACAGCGCAATCTACTTCAAGAGTCCTTATCGACAAGTGGGTTTACGACCTCGATGTTGGATCAGGGTACCCCAGTGGTGCAGCCGCTACTAAAGGTTCGTTTGTTCAACGATTAAAACCCTACGTGATCTGAGTTCAGACCGGA

>Seq4 [organism-Scinax x-sigantus] Scinax x-sigantus 16S ribosomal RNA, partial cds; mitochondrial.

CCGCgGTATCCTAACCGTGCGAAGGTAGCGTAATCACTTGTTCTTTAAATGAGGACTAGTATGAATGGCACCACGAGGGTTGCACTGTCTCCTTCCCCTAATCAATGAAACTAATCTTCCCGTGAAGAAGCGGGAATTTATTTATAAGACGAGAAGACCCTATGGAGCTTTAAATTTCTCTTCAAACGCCAGACATAAACCCTAAACATCTTACACCAACCCGGCGCCATGAAGATGAATTTTAGGTTGGGGTGACCACGGAGAACAAGACAACCTCCATGCTGAATAGGCCTAAAACCTTGAACTAAGGACCACAGCCCGACGAACTAAAAAATTAACACCCATTGACCCAATTCTTTTTGATCAACGAACCAAGTTACCCTAGGGATAACAGCGCAATCTACTTCAAGAGTCCTTATCGACAAGTGGGTTTACGACCTCGATGTTGGATCAGGGTACCCCAGTGGTGCAGCCGCTACTAAAGGTTCGTTTGTTCAACGATTAAAACCCTACGTGATCTGAGTTCAGACCGGA

>Seq5 [organism-Scinax x-sigantus] Scinax x-sigantus 16S ribosomal RNA, partial cds; mitochondrial.

CCGcGGTATCCTAACCGTGCGAAGGtAGCGTAATCACTTGTTCTTTAAATGAGGACTAGTATGAATGGCACCACGAGGGTTGCACTGTCTCCTCCCCCTAATCAATGAAACTAATCTTCCCGTGAAGAAGCGGGAATTTATTTATAAGACGAGAAGACCCTATGGAGCTTTAAATTTCTCTTCAAACGCCAGACATAAACCCTAAACATCTTACACCAACCCGGCGCCATGAAGATGAATTTTAGGTTGGGGTGACCACGGAGAACAAGACAACCTCCATGCTGAATAGGCCTAAAACCTTGAACTAAGGACCACAGCCCGACGGACTAAAAAATTAACACCCATTGACCCAATTCTTTTTGATCAACGAACCAAGTTACCCTAGGGATAACAGCGCAATCTACTTCAAGAGTCCTTATCGACAAGTGGGTTTACGACCTCGATGTTGGATCAGGGTACCCCAGTGGTGCAGCCGCTACTAAAGGTTCGTTTGTTCAACGATTAAAACCCTACGTGATCTGAGTTCAGACCGGA

>Seq1 [organism-Pithecopus aff. hypochondrialis] Pithecopus aff. hypochondrialis 16S ribosomal RNA, partial cds; mitochondrial.

CCGCGGTATCCTAACCGTGCGAAGGTAGCGTAATCACTTGTTCTTTAAATGAGGACTAGTATGAATGGCATTACGAGGGTTATACTGTCTCCTTTTCCTAATCAGTGAAACTAATCTCCCCGTGAAGAAGCGAGGATGAACATATAAGACGAGAAGACCCTATGGAGCTTTAAACTTCCAGCAATTATCTTTTTTATACCAACCTCAAGGTAATCAAATTAACTTTAATACTATGTCTGTTAGTTTTCGGTTGGGGTGACCGCGGAGTAAAACTCACCCTCCGCGATGAATACTAAACATCTAAGCAACAAACTACAATTTTACGCATCAAAAAATTGACACTATTGACCCAACATTTTGATCAACGAACCAAGTTACCCTAGGGATAACAGCGCAATCCATCTCAAGAGCTCATATCGACAGGTGGGTTTACGACCTCGATGTTGGATCAGGATATCCCAGTGGTGCAGCCGCTACTAAAGGTTCGTTTGTTCAACGATTAAAATCCTACGTGATCTGAGTTCAGACCGGA

>Seq2 [organism-Pithecopus aff. hypochondrialis] Pithecopus aff. hypochondrialis 16S ribosomal RNA, partial cds; mitochondrial.

CCGCGGTATCCTAACCGTGCGAAGGTAGCGTAATCACTTGTTCTTTAAATGAGGACTAGTATGAATGGCATTACGAGGGTTATACTGTCTCCTTTTCCTAATCAGTGAAACTAATCTCCCCGTGAAGAAGCGAGGATGAACATATAAGACGAGAAGACCCTATGGAGCTTTAAACTTCCAGCAATTATCTTTTTTATACCAACCTCAAGGTAATCAAATTAACTTTAATACTATGTCTGTTAGTTTTCGGTTGGGGCGACCGCGGAGTAAAACTCACCCTCCGCGATGAATACTAAACATCTAAGCAACAAACTACAATTTTACGCATCAAAAAATTGACACTATTGACCCAACATTTTGATCAACGAACCAAGTTACCCTAGGGATAACAGCGCAATCCATCTCAAGAGCTCATATCGACAGGTGGGTTTACGACCTCGATGTTGGATCAGGATATCCCAGTGGTGCAGCCGCTACTAAAGGTTCGTTTGTTCAACGATTAAAATCCTACGTGATCTGAGTTCAGACCGGA

>Seq3 [organism-Pithecopus aff. hypochondrialis] Pithecopus aff. hypochondrialis 16S ribosomal RNA, partial cds; mitochondrial.

CCGCGGTATCCTAACCGTGCGAAGGTAGCGTAATCACTTGTTCTTTAAATGAGGACTAGTATGAATGGCATTACGAGGGTTATACTGTCTCCTTTTCCTAATCAGTGAAACTAATCTCCCCGTGAAGAAGCGAGGATGAACATATAAGACGAGAAGACCCTATGGAGCTTTAAACTTCCAGCAATTATCTTTTTTATACCAACCTCAAGGTAATCAAATTAACTTTAATACTATGTCTGTTAGTTTTCGGTTGGGGTGACCGCGGAGTAAAACTCACCCTCCGCGATGAATACTAAATATCTAAGCAACAAACTACAATTTTACGCATCAAAAAATTGACACTATTGACCCAACATTTTGATCAACGAACCAAGTTACCCTAGGGATAACAGCGCAATCCATCTCAAGAGCTCATATCGACAGGTGGGTTTACGACCTCGATGTTGGATCAGGATATCCCAGTGGTGCAGCCGCTACTAAAGGTTCGTTTGTTCAACGATTAAAATCCTACGTGATCTGAGTTCAGACCGGA

>Seq4 [organism-Pithecopus aff. hypochondrialis] Pithecopus aff. hypochondrialis 16S ribosomal RNA, partial cds; mitochondrial.

CCGCGGTATCCTAACCGTGCGAAGGTAGCGTAATCACTTGTTCTTTAAATGAGGACTAGTATGAATGGCATTACGAGGGTTATACTGTCTCCTTTTCCTAATCAGTGAAACTAATCTCCCCGTGAAGAAGCGAGGATGAACATATAAGACGAGAAGACCCTATGGAGCTTTAAACTTCCAGCAATTATCTTTTTTATACCAACCTCAAGGTAATCAAATTAACTTTAATACTATGTCTGTTAGTTTTCGGTTGGGGCGACCGCGGAGTAAAACTCACCCTCCGCGATGAATACTAAACATCTAAGCAACAAACTACAATTTTACGCATCAAAAAATTGACACTATTGACCCAACATTTTGATCAACGAACCAAGTTACCCTAGGGATAACAGCGCAATCCATCTCAAGAGCTCATATCGACAGGTGGGTTTACGACCTCGATGTTGGATCAGGATATCCCAGTGGTGCAGCCGCTACTAAAGGTTCGTTTGTTCAACGATTAAAATCCTACGTGATCTGAGTTCAGACCGGA

>Seq5 [organism-Pithecopus aff. hypochondrialis] Pithecopus aff. hypochondrialis 16S ribosomal RNA, partial cds; mitochondrial.

CCGCGGTATCCTAACCGTGCGAAGGTAGCGTAATCACTTGTTCTTTAAATGAGGACTAGTATGAATGGCATTACGAGGGTTATACTGTCTCCTTTTCCTAATCAGTGAAACTAATCTCCCCGTGAAGAAGCGAGGATGAACATATAAGACGAGAAGACCCTATGGAGCTTTAAACTTCCAGCAATTATCTTTTTTATACCAACCTCAAGGCAATCAAATTAACTTTAATACTATGTCTGTTAGTTTTCGGTTGGGGTGACCGCGGAGTAAAACTCACCCTCCGCGATGAATACTAAACATCTAAGCAACAAACTACAATTTTACGCATCAAAAAATTGACACTATTGACCCAACATTTTGATCAACGAACCAAGTTACCCTAGGGATAACAGCGCAATCCATCTCAAGAGCTCATATCGACAGGTGGGTTTACGACCTCGATGTTGGATCAGGATATCCCAGTGGTGCAGCCGCTACTAAAGGTTCGTTTGTTCAACGATTAAAATCCTACGTGATCTGAGTTCAGACCGGA
