# Supplementary material for: Discovering the diversity of tadpoles in the mid-north Brazil: morphological and molecular identification, and characterization of the habitat
Source: PeerJ. 2023 Dec 14;11:e16640. doi: 10.7717/peerj.16640 (PMC10725668; doi:10.7717/peerj.16640)
Supplement: Supplemental Information 5 [file peerj-11-16640-s005.docx]

## Discovering the diversity of tadpoles in the mid-north Brazil: morphological and molecular identification, and characterization of the habitat

Patrícia dos Santos Sousa^1^, Carlos Augusto Silva de Azevêdo^1^, Maria Claudene Barros^1^, Elmary da Costa Fraga^1^, Thaís B. Guedes^2,3^

^1^Centro de Estudos Superiores de Caxias, Universidade Estadual do Maranhão, 65604-380, Caxias, MA, Brazil

^2^Departamento de Biologia Animal, Instituto de Biologia, Universidade Estadual de Campinas, 13083-862, Campinas, SP, Brazil

^3^Gothenburg Global Biodiversity Center, University of Gothenburg, Department of Biological and Environmental Sciences, Box 461, SE-405-30, Göteborg, Sweden

Corresponding author: Thaís B. Guedes. Address: Rua Monteiro Lobato, 255, Cidade Universitária, 13083-862, Campinas, SP, Brazil. E-mail: thaisbguedes@yahoo.com.br

Supporting information

**Appendix S5.** GenBank accession numbers for the gene rRNA 16S used as reference library in the present study.

| Species | GenBank numbers | | |
| --- | --- | --- | --- |
| **Bufonidae** |  | | |
| *Rhinella diptycha* (*R*. *jimi*) | MH004313 | | |
| *Rhinella mirandaribeiroi* | KP685226 | | |
| **Hylidae** |  | | |
| *Boana multifasciata* | OP037631 | | |
| *Boana* cf. *atlantica* | MK348503 | | |
| *Dendropsophus soaresi* | MT503922 | | |
| *Osteocephalus taurinus* | JX564881 | | |
| *Pithecopus aff. hypochondrialis* | KC520707 | | |
|  |  | | |
| *Scinax x-signatus* | | OP022082 |  |
| *Scinax fuscomarginatus* | | KJ004150 |  |
| *Scinax nebulosus* | | KJ004190 |  |
| *Scinax cf. similis* | | OP022101 |  |
| *Trachycephalus typhonius* | | KF723121 |  |
| **Leptodactylidae (Leiuperinae)** | |  |  |
| *Physalaemus cuvieri* | | HQ592351 |  |
| *Physalaemus nattereri* | | JQ627214 |  |
| **Leptodactylidae (Leptodactylinae)** | |  |  |
| *Leptodactylus fuscus* | | AY911281 |  |
| *Leptodactylus macrosternum* | | MT495861 |  |
| *Leptodactylus mystaceus* | | MN958079 |  |
| *Leptodactylus natalensis* | | MH004304 |  |
| *Leptodactylus pustulatus* | | MW291413 |  |
| *Leptodactylus troglodytes* | | KM091620 |  |
| **Microhylidae (Gastrophryninae)** | |  |  |
| *Dermatonotus muelleri* | | MH004297 |  |
| *Elachistocleis cesarii* | | KM509129 |  |
