# Supplementary material for: Discovering the diversity of tadpoles in the mid-north Brazil: morphological and molecular identification, and characterization of the habitat
Source: PeerJ. 2023 Dec 14;11:e16640. doi: 10.7717/peerj.16640 (PMC10725668; doi:10.7717/peerj.16640)
Supplement: Supplemental Information 7 — The numbers on the diagonal, in bold, correspond to the intrapopulation mean. [file peerj-11-16640-s007.docx]

## Discovering the diversity of tadpoles in the mid-north Brazil: morphological and molecular identification, and characterization of the habitat

Patrícia dos Santos Sousa^1^, Carlos Augusto Silva de Azevêdo^1^, Maria Claudene Barros^1^, Elmary da Costa Fraga^1^, Thaís B. Guedes^2,3^

^1^Centro de Estudos Superiores de Caxias, Universidade Estadual do Maranhão, 65604-380, Caxias, MA, Brazil

^2^Departamento de Biologia Animal, Instituto de Biologia, Universidade Estadual de Campinas, 13083-862, Campinas, SP, Brazil

^3^Gothenburg Global Biodiversity Center, University of Gothenburg, Department of Biological and Environmental Sciences, Box 461, SE-405-30, Göteborg, Sweden

Corresponding author: Thaís B. Guedes. Address: Rua Monteiro Lobato, 255, Cidade Universitária, 13083-862, Campinas, SP, Brazil. E-mail: thaisbguedes@yahoo.com.br

**Supporting information**

**Appendix S7.** Percentage of nucleotide divergences generated for the gene rRNA 16S for the tadpole species in the present study. The numbers on the diagonal, in bold, correspond to the intrapopulation mean.

| **SPECIES** | **GENETIC DISTANCE (%)** | | | | | | | | | | | | | | | | | | | | | | |
| --- | --- | --- | --- | --- | --- | --- | --- | --- | --- | --- | --- | --- | --- | --- | --- | --- | --- | --- | --- | --- | --- | --- | --- |
|  | **1** | **2** | **3** | **4** | **5** | **6** | **7** | **8** | **9** | **10** | **11** | **12** | **13** | **14** | **15** | **16** | **17** | **18** | **19** | **20** | **21** | **22** | **23** |
| 1. *B*. *multifasciata* | **0.23** |  |  |  |  |  |  |  |  |  |  |  |  |  |  |  |  |  |  |  |  |  |  |
| 2. *B*. cf. *punctata* | 17.70 | **0** |  |  |  |  |  |  |  |  |  |  |  |  |  |  |  |  |  |  |  |  |  |
| 3. *O*. *taurinus* | 20.04 | 19.61 | **0.38** |  |  |  |  |  |  |  |  |  |  |  |  |  |  |  |  |  |  |  |  |
| 4. *D*. *soaresi* | 22.51 | 20.36 | 13.94 | **0.15** |  |  |  |  |  |  |  |  |  |  |  |  |  |  |  |  |  |  |  |
| 5. *T*. *typhonius* | 17.15 | 18.37 | 8.34 | 11.88 | **0.39** |  |  |  |  |  |  |  |  |  |  |  |  |  |  |  |  |  |  |
| 6. *S*. *fuscomarginatus* | 25.19 | 24.89 | 21.33 | 25.32 | 22.04 | **0.10** |  |  |  |  |  |  |  |  |  |  |  |  |  |  |  |  |  |
| 7. *S*. cf. *nebulosus* | 24.87 | 23.56 | 18.35 | 21.80 | 18.92 | 20.90 | **0.26** |  |  |  |  |  |  |  |  |  |  |  |  |  |  |  |  |
| 8. *S*. cf. *similis* | 22.61 | 21.53 | 18.81 | 24.54 | 20.73 | 17.27 | 18.55 | **0.13** |  |  |  |  |  |  |  |  |  |  |  |  |  |  |  |
| 9. *S*. *x-signatus* | 24.84 | 20.91 | 21.52 | 22.75 | 22.41 | 17.17 | 17.94 | 12.36 | **0.43** |  |  |  |  |  |  |  |  |  |  |  |  |  |  |
| 10. *P*. *nattereri* | 20.32 | 24.05 | 17.28 | 20.55 | 16.43 | 25.39 | 24.62 | 24.68 | 24.77 | **0.50** |  |  |  |  |  |  |  |  |  |  |  |  |  |
| 11. *P*. *cuvieri* | 23.61 | 21.76 | 17.20 | 22.82 | 17.08 | 25.33 | 25.79 | 24.34 | 24.78 | 11.49 | **0.50** |  |  |  |  |  |  |  |  |  |  |  |  |
| 12. *L*. *fuscus* | 19.04 | 19.12 | 16.16 | 17.10 | 15.22 | 25.62 | 19.83 | 20.86 | 21.02 | 19.53 | 18.09 | **0.95** |  |  |  |  |  |  |  |  |  |  |  |
| 13. *L*. *pustulatus* | 21.88 | 25.73 | 17.28 | 22.21 | 18.00 | 26.49 | 23.36 | 21.95 | 20.36 | 20.27 | 21.13 | 12.01 | **0.80** |  |  |  |  |  |  |  |  |  |  |
| 14. *L*. *macrosternum* | 20.72 | 22.68 | 18.48 | 22.06 | 15.62 | 27.49 | 22.34 | 22.58 | 22.41 | 18.63 | 18.30 | 12.24 | 9.61 | **0.58** |  |  |  |  |  |  |  |  |  |
| 15. *L*. *troglodytes* | 21.36 | 21.18 | 17.92 | 23.16 | 17.70 | 26.08 | 22.02 | 21.89 | 20.10 | 21.76 | 18.34 | 8.47 | 12.67 | 12.64 | **0.20** |  |  |  |  |  |  |  |  |
| 16. *L*. *natalensis* | 23.19 | 25.04 | 19.99 | 23.17 | 18.04 | 26.46 | 21.93 | 20.20 | 18.86 | 20.98 | 20.47 | 13.05 | 12.25 | 12.77 | 13.51 | **1.09** |  |  |  |  |  |  |  |
| 17. *L*. *mystaceus* | 21.11 | 23.63 | 19.19 | 23.62 | 17.45 | 25.71 | 20.46 | 24.09 | 23.32 | 20.26 | 21.82 | 9.21 | 15.02 | 13.14 | 13.26 | 15.52 | **0.82** |  |  |  |  |  |  |
| 18. *R*. *mirandaribeiroi* | 21.67 | 17.93 | 18.55 | 21.16 | 16.55 | 22.45 | 22.99 | 21.30 | 22.82 | 18.70 | 17.67 | 17.68 | 20.33 | 18.87 | 21.10 | 21.45 | 22.42 | **0.55** |  |  |  |  |  |
| 19. *R*. *diptycha* | 23.63 | 20.57 | 19.99 | 22.76 | 19.20 | 23.42 | 23.03 | 23.89 | 23.69 | 21.17 | 20.89 | 17.58 | 18.58 | 18.30 | 19.83 | 22.47 | 21.31 | 8.87 | **0.19** |  |  |  |  |
| 20. *D*. *muelleri* | 24.50 | 27.61 | 19.74 | 23.03 | 19.29 | 25.89 | 25.53 | 21.84 | 23.78 | 18.88 | 19.40 | 20.19 | 21.84 | 19.65 | 19.06 | 22.82 | 21.95 | 21.86 | 21.18 | **0.20** |  |  |  |
| 22. *E*. *cesarii* | 22.03 | 23.70 | 19.02 | 23.95 | 19.09 | 25.98 | 24.72 | 22.21 | 23.62 | 17.79 | 18.45 | 20.84 | 22.93 | 21.00 | 21.41 | 20.07 | 24.72 | 18.26 | 17.77 | 11.63 | **2.31** |  |  |
| 23. *P*. aff. *hypochodrialis* | 22.03 | 21.98 | 16.79 | 19.19 | 17.10 | 24.82 | 24.75 | 25.06 | 26.03 | 23.27 | 23.52 | 20.51 | 22.41 | 22.27 | 22.91 | 25.69 | 24.37 | 22.29 | 21.74 | 24.02 | 21.60 | **0.49** |  |
| Outgroup | 31.08 | 33.20 | 26.24 | 30.77 | 27.82 | 34.09 | 35.30 | 35.29 | 33.76 | 31.23 | 31.93 | 26.70 | 29.69 | 29.46 | 29.02 | 30.95 | 30.54 | 30.87 | 29.64 | 34.10 | 34.85 | 32.58 | **30.0** |
