# Supplementary material for: Discovering the diversity of tadpoles in the mid-north Brazil: morphological and molecular identification, and characterization of the habitat
Source: PeerJ. 2023 Dec 14;11:e16640. doi: 10.7717/peerj.16640 (PMC10725668; doi:10.7717/peerj.16640)
Supplement: Supplemental Information 8 — Molecular identification was performed by comparing the sequences generated in this study and those provided in the BLAST platform. [file peerj-11-16640-s008.docx]

## Discovering the diversity of tadpoles in the mid-north Brazil: morphological and molecular identification, and characterization of the habitat

Patrícia dos Santos Sousa^1^, Carlos Augusto Silva de Azevêdo^1^, Maria Claudene Barros^1^, Elmary da Costa Fraga^1^, Thaís B. Guedes^2,3^

^1^Centro de Estudos Superiores de Caxias, Universidade Estadual do Maranhão, 65604-380, Caxias, MA, Brazil

^2^Departamento de Biologia Animal, Instituto de Biologia, Universidade Estadual de Campinas, 13083-862, Campinas, SP, Brazil

^3^Gothenburg Global Biodiversity Center, University of Gothenburg, Department of Biological and Environmental Sciences, Box 461, SE-405-30, Göteborg, Sweden

Corresponding author: Thaís B. Guedes. Address: Rua Monteiro Lobato, 255, Cidade Universitária, 13083-862, Campinas, SP, Brazil. E-mail: thaisbguedes@yahoo.com.br

Supporting information

**Appendix S8.** Comparison between the results from morphological characterization and molecular identification. Molecular identification was performed by comparing the sequences generated in this study and those provided in the BLAST platform. GFN: Guedes Field Number

| **GFN - Tadpoles** | **Morphological characterization** | **Molecular identification** | **Similarity from BLAST** |
| --- | --- | --- | --- |
| 71 | *Rhinella diptycha* | *R*. *diptycha* | 99.47% |
| 72 | *Rhinella diptycha* | *R*. *diptycha* | 99.47% |
| 73 | *Rhinella diptycha* | *R*. *diptycha* | 99.47% |
| 77 | *Rhinella diptycha* | *R*. *diptycha* | 99.29% |

| 78 | *Rhinella diptycha* | *R*. *diptycha* | 95.74% |
| --- | --- | --- | --- |
| 149 | *Rhinella* cf. *mirandaribeiroi* | *R*. *mirandaribeiroi* | 98.51% |
| 147 | *Rhinella* cf. *mirandaribeiroi* | *R*. *mirandaribeiroi* | 98.72% |
| 146 | *Rhinella* cf. *mirandaribeiroi* | *R*. *mirandaribeiroi* | 98.72% |
| 185 | *Rhinella* cf. *mirandaribeiroi* | *R*. *mirandaribeiroi* | 98.75% |
| 186 | *Rhinella* cf. *mirandaribeiroi* | *R*. *mirandaribeiroi* | 98.93% |
| 36 | *Boana* sp. | *B*. *multifasciata* | 99.82% |
| 07 | *Boana* sp. | *B*. *multifasciata* | 99.63% |
| 164 | *Boana* sp. | *B*. *multifasciata* | 99.45% |
| 165 | *Boana* sp. | *B*. *multifasciata* | 99.63% |
| 81 | *Boana* sp. | *B*. *multifasciata* | 99.82% |
| 82 | *Boana* sp. | *B*. *multifasciata* | 99.08% |
| 88 | *Boana punctata* | *B.* cf. *atlantica* | 100% |
| 89 | *Boana punctata* | *B.* cf. *atlantica* | 100% |
| 93 | *Boana punctata* | *B.* cf. *atlantica* | 100% |
| 94 | *Boana punctata* | *B.* cf. *atlantica* | 98.94% |
| 95 | *Boana punctata* | *B.* cf. *atlantica* | 98.86% |
| 177 | *Dendropsophus* cf. *melanargyreus* | *D*. *soaresi* | 99.64% |
| 13 | *Dendropsophus* cf. *melanargyreus* | *D. soaresi* | 99.81% |
| 25 | *Dendropsophus* sp. | *D. soaresi* | 100% |

| 169 | *Dendropsophus* cf. *melanargyreus* | *D*. *soaresi* | 99.46% |
| --- | --- | --- | --- |
| 170 | *Dendropsophus* cf. *melanargyreus* | *D*. *soaresi* | 99.45% |
| 171 | *Dendropsophus* cf. *melanargyreus* | *D*. *soaresi* | 99.45% |
| 172 | *Dendropsophus* cf. *melanargyreus* | *D*. *soaresi* | 99.28% |
| 173 | *Dendropsophus* cf. *melanargyreus* | *D*. *soaresi* | 99.45% |
| 175 | *Dendropsophus* cf. *melanargyreus* | *D*. *soaresi* | 99.82% |
| 176 | *Dendropsophus* cf. *melanargyreus* | *D*. *soaresi* | 98.39% |
| 18 | *Osteocephalus taurinus* | *O*. *taurinos* | 99.13% |
| 19 | *Osteocephalus taurinus* | *O*. *taurinos* | 99.13% |
| 34 | *Osteocephalus taurinus* | *O*. *taurinos* | 99.48% |
| 35 | *Osteocephalus taurinus* | *O*. *taurinos* | 99.96% |
| 37 | *Osteocephalus taurinus* | *O*. *taurinos* | 99.48% |
| 38 | *Osteocephalus taurinus* | *O*. *taurinos* | 99.14% |
| 39 | *Osteocephalus taurinus* | *O*. *taurinos* | 99.97% |
| 160 | *Scinax* sp. 1 | *S*. *x-signatus* | 99.44% |
| 24 | *Scinax* sp. 1 | *S*. *x-signatus* | 100.00% |
| 161 | *Scinax* sp. 1 | *S*. *x-signatus* | 99.24% |
| 190 | *Scinax* sp. 1 | *S*. *x-signatus* | 98.50% |
| 202 | *Scinax* sp. 1 | *S*. *x-signatus* | 99.44% |
| 180 | *Scinax* sp. 1 | *S*. *nebulosus* | 98.74% |

| 201 | *Scinax* sp. 1 | *S*. *nebulosus* | 99.64% |
| --- | --- | --- | --- |
| 189 | *Scinax* cf. *fuscomarginatus* | *S. fuscomarginatus* | 99.81% |
| 200 | *Scinax* cf. *fuscomarginatus* | *S. fuscomarginatus* | 99.45% |
| 199 | *Scinax* cf. *fuscomarginatus* | *S. fuscomarginatus* | 97.86% |
| 40 | *Scinax* sp. 2 | *S*. cf. *similis* | 99.82% |
| 44 | *Scinax* sp. 2 | *S*. cf. *similis* | 99.82% |
| 45 | *Scinax* sp. 2 | *S*. cf. *similis* | 99.64% |
| 46 | *Scinax* sp. 2 | *S*. cf. *similis* | 99.82% |
| 11 | *Scinax* sp. 2 | *S*. cf. *similis* | 99.82% |
| 12 | *Scinax* sp. 2 | *S*. cf. *similis* | 99.82% |
| 14 | *Scinax* sp. 2 | *S*. cf. *similis* | 99.82% |
| 124 | *Trachycephalus typhonius* | *T*. *typhonius* | 98.23% |
| 125 | *Trachycephalus typhonius* | *T*. *typhonius* | 99.08% |
| 126 | *Trachycephalus typhonius* | *T*. *typhonius* | 98.23% |
| 127 | *Trachycephalus typhonius* | *T*. *typhonius* | 99.45% |
| 128 | *Trachycephalus typhonius* | *T*. *typhonius* | 99.29% |
| 54 | *Physalaemus cuvieri* | *P*. *cuvieri* | 99.07% |
| 55 | *Physalaemus cuvieri* | *P*. *cuvieri* | 99.09% |
| 58 | *Physalaemus cuvieri* | *P*. *cuvieri* | 99.08% |
| 60 | *Physalaemus cuvieri* | *P*. *cuvieri* | 98.88% |

| 20 | *Physalaemus cuvieri* | *P*. *cuvieri* | 98.72% |
| --- | --- | --- | --- |
| 21 | *Physalaemus cuvieri* | *P*. *cuvieri* | 99.08% |
| 22 | *Physalaemus cuvieri* | *P*. *cuvieri* | 98.34% |
| 23 | *Physalaemus cuvieri* | *P*. *cuvieri* | 99.08% |
| 143 | *Physalaemus cuvieri* | *P*. *cuvieri* | 99.07% |
| 142 | *Physalaemus cuvieri* | *P*. *cuvieri* | 98.15% |
| 140 | *Physalaemus cuvieri* | *P*. *cuvieri* | 98.35% |
| 84 | *Physalaemus* cf. *nattereri* | *P*. *nattereri* | 97.92% |
| 106 | *Physalaemus* cf. *nattereri* | *P*. *nattereri* | 98.12% |
| 153 | *Physalaemus* cf. *nattereri* | *P*. *nattereri* | 98.96% |
| 182 | *Physalaemus* cf. *nattereri* | *P*. *nattereri* | 98.53% |
| 183 | *Physalaemus* cf. *nattereri* | *P*. *nattereri* | 98.53% |
| 184 | *Physalaemus* cf. *nattereri* | *P*. *nattereri* | 98.50% |
| 105 | *Leptodactylus fuscus* | *L*. *fuscus* | 98.44% |
| 107 | *Leptodactylus fuscus* | *L*. *fuscus* | 98.45% |
| 108 | *Leptodactylus fuscus* | *L*. *fuscus* | 98.79% |
| 110 | *Leptodactylus fuscus* | *L*. *fuscus* | 98.26% |
| 129 | *Leptodactylus macrosternum* | *L*. *macrosternum* | 98.73% |
| 130 | *Leptodactylus macrosternum* | *L*. *macrosternum* | 98.91% |
| 131 | *Leptodactylus macrosternum* | *L*. *macrosternum* | 99.27% |

| 132 | *Leptodactylus macrosternum* | *L*. *macrosternum* | 99.45% |
| --- | --- | --- | --- |
| 133 | *Leptodactylus macrosternum* | *L*. *macrosternum* | 99.08% |
| 114 | *Leptodactylus mystaceus* | *L*. *mystaceus* | 97.65% |
| 115 | *Leptodactylus mystaceus* | *L*. *mystaceus* | 98.21% |
| 116 | *Leptodactylus mystaceus* | *L*. *mystaceus* | 97.14% |
| 117 | *Leptodactylus mystaceus* | *L*. *mystaceus* | 96.99% |
| 187 | *Leptodactylus mystaceus* | *L*. *mystaceus* | 97.68% |
| 188 | *Leptodactylus mystaceus* | *L*. *mystaceus* | 98.03% |
| 15 | *Leptodactylus natalensis* | *L*. *natalensis* | 99.63% |
| 16 | *Leptodactylus natalensis* | *L*. *natalensis* | 99.63% |
| 17 | *Leptodactylus natalensis* | *L*. *natalensis* | 99.64% |
| 47 | *Leptodactylus natalensis* | *L*. *natalensis* | 99.45% |
| 48 | *Leptodactylus natalensis* | *L*. *natalensis* | 99.45% |
| 49 | *Leptodactylus natalensis* | *L*. *natalensis* | 97.62% |
| 50 | *Leptodactylus natalensis* | *L*. *natalensis* | 99.63% |
| 51 | *Leptodactylus natalensis* | *L*. *natalensis* | 99.64% |
| 52 | *Leptodactylus natalensis* | *L*. *natalensis* | 98.54% |
| 191 | *Leptodactylus natalensis* | *L*. *natalensis* | 100% |
| 195 | *Leptodactylus natalensis* | *L*. *natalensis* | 99.80% |
| 194 | *Leptodactylus natalensis* | *L*. *natalensis* | 99.63% |

| 193 | *Leptodactylus natalensis* | *L*. *natalensis* | 99.45% |
| --- | --- | --- | --- |
| 192 | *Leptodactylus natalensis* | *L*. *natalensis* | 99.45% |
| 63 | *Leptodactylus* cf. *pustulatus* | *L*. *pustulatus* | 98.17% |
| 64 | *Leptodactylus* cf. *pustulatus* | *L*. *pustulatus* | 99.09% |
| 65 | *Leptodactylus* cf. *pustulatus* | *L*. *pustulatus* | 97.45% |
| 67 | *Leptodactylus* cf. *pustulatus* | *L*. *pustulatus* | 98.88% |
| 66 | *Leptodactylus* cf. *pustulatus* | *L*. *pustulatus* | 98.69% |
| 01 | *Leptodactylus troglodytes* | *L*. *troglodytes* | 98.75% |
| 02 | *Leptodactylus troglodytes* | *L*. *troglodytes* | 99.10% |
| 03 | *Leptodactylus troglodytes* | *L*. *troglodytes* | 98.74% |
| 04 | *Leptodactylus troglodytes* | *L*. *troglodytes* | 99.28% |
| 05 | *Leptodactylus troglodytes* | *L*. *troglodytes* | 99.28% |
| 06 | *Leptodactylus troglodytes* | *L*. *troglodytes* | 99.45% |
| 83 | *Dermatonotus muelleri* | *D*. *muelleri* | 98.05% |
| 163 | *Elachistocleis* sp. | *E*. *cesarii* | 98.10% |
| 08 | *Pithecopus hypochondrialis* | *P*. *hypochondrialis* | 99.10% |
| 09 | *Pithecopus hypochondrialis* | *P*. aff. *hypochondrialis* | 98.58% |
| 10 | *Pithecopus hypochondrialis* | *P*. aff. *hypochondrialis* | 99.29% |
| 29 | *Pithecopus hypochondrialis* | *P*. aff. *hypochondrialis* | 98.94% |
| 30 | *Pithecopus hypochondrialis* | *P*. aff*. hypochondrialis* | 98.76% |

| 31 | *Pithecopus hypochondrialis* | *P*. aff*. hypochondrialis* | 98.60% |
| --- | --- | --- | --- |
| 32 | *Pithecopus hypochondrialis* | *P*. aff. *hypochondrialis* | 98.94% |
| 33 | *Pithecopus hypochondrialis* | *P*. aff. *hypochondrialis* | 98.77% |
