# Supplementary material for: Discovering the diversity of tadpoles in the mid-north Brazil: morphological and molecular identification, and characterization of the habitat
Source: PeerJ. 2023 Dec 14;11:e16640. doi: 10.7717/peerj.16640 (PMC10725668; doi:10.7717/peerj.16640)
Supplement: Supplemental Information 9 — The short, paired semicircular dermal flaps in front of the mouth in the first photo and long semicircular and significantly exceeding the front of the mouth in the second photograph [file peerj-11-16640-s009.docx]

## Discovering the diversity of tadpoles in the mid-north Brazil: morphological and molecular identification, and characterization of the habitat

Patrícia dos Santos Sousa^1^, Carlos Augusto Silva de Azevêdo^1^, Maria Claudene Barros^1^, Elmary da Costa Fraga^1^, Thaís B. Guedes^2,3^

^1^Centro de Estudos Superiores de Caxias, Universidade Estadual do Maranhão, 65604-380, Caxias, MA, Brazil

^2^Departamento de Biologia Animal, Instituto de Biologia, Universidade Estadual de Campinas, 13083-862, Campinas, SP, Brazil

^3^Gothenburg Global Biodiversity Center, University of Gothenburg, Department of Biological and Environmental Sciences, Box 461, SE-405-30, Göteborg, Sweden

Corresponding author: Thaís B. Guedes. Address: Rua Monteiro Lobato, 255, Cidade Universitária, 13083-862, Campinas, SP, Brazil. E-mail: thaisbguedes@yahoo.com.br

**Supporting Information**


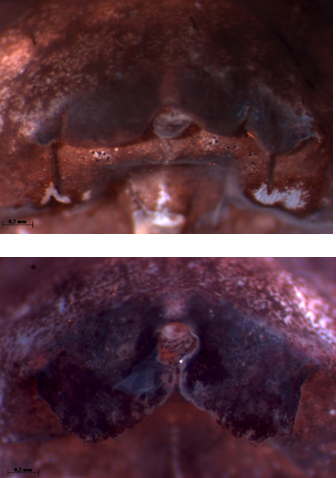


**Appendix S9.** Variation in the shape of the dermal flap of *Elachistocleis cesarii*, the first photo (up) at stage 35 of Gosner (1960), the second was observed at stages 34 to 36 (down). The short, paired semicircular dermal flaps in front of the mouth in the first photo and long semicircular and significantly exceeding the front of the mouth in the second photograph.
